# Uncovering the Underlying Mechanisms Blocking Replication of Bluetongue Virus Serotype 26 (BTV-26) in *Culicoides* Cells

**DOI:** 10.3390/biom13060878

**Published:** 2023-05-23

**Authors:** Baptiste Monsion, Fauziah Mohd Jaafar, Peter P. C. Mertens, Houssam Attoui

**Affiliations:** 1 UMR1161 VIROLOGIE, INRAE, Ecole Nationale Vétérinaire d’Alfort, ANSES, Université Paris-Est, F-94700 Maisons-Alfort, France; 2 One Virology, The Wolfson Centre for Global Virus Research, School of Veterinary Medicine and Science, University of Nottingham, Sutton Bonington Campus, Loughborough LE12 5RD, Leicestershire, UK

**Keywords:** bluetongue virus, atypical bluetongue virus, BTV-26, BTV-1, blocked replication, insect cells, horizontal transmission, mechanisms underlying blocking replication

## Abstract

At least 12 serotypes of ‘atypical’ bluetongue virus (BTV-25 to BTV-36) have been identified to date. These atypical serotypes fail to infect/replicate in *Culicoides*-derived cell lines and/or adult *Culicoides* vectors and hence can no longer be transmitted by these vectors. They appear to be horizontally transmitted from infected to in-contact ruminants, although the route(s) of infection remain to be identified. Viral genome segments 1, 2 and 3 (Seg-1, Seg2 and Seg-3) of BTV-26 were identified as involved in blocking virus replication in KC cells. We have developed *Culicoides*-specific expression plasmids, which we used in transfected insect cells to assess the stability of viral mRNAs and protein expression from full-length open reading frames of Seg-1, -2 and -3 of BTV-1 (a *Culicoides*-vectored BTV) or BTV-26. Our results indicate that the blocked replication of BTV-26 in KC cells is not due to an RNAi response, which would lead to rapid degradation of viral mRNAs. A combination of degradation/poor expression and/or modification of the proteins encoded by these segments appears to drive the failure of BTV-26 core/whole virus-particles to assemble and replicate effectively in *Culicoides* cells.

## 1. Introduction

There are twenty-two officially recognised species within the genus *Orbivirus* (family *Sedoreoviridae,* order *Reovirales*), with *Bluetongue virus* as the ‘type-species’. However, recent studies suggest the existence of several additional species [1,2]. Orbiviruses are vector-borne viruses (arboviruses), that are mainly transmitted between susceptible hosts by the bite of hematophagous arthropods, including *Culicoides* midges, ticks, phlebotomine flies, anopheline or culicine mosquitoes, in which they also replicate [3,4]. Collectively, they can infect a wide range of vertebrate hosts, including ruminants, equids, humans, marsupials, birds and reptiles. Three economically important orbiviruses are transmitted by *Culicoides* biting-midges, including *Bluetongue virus* (BTV), *African horse sickness virus* (AHSV) and *Epizootic hemorrhagic disease virus* (EHDV) [5].

Until 2008, twenty-four BTV serotypes were recognised, which were all thought to be transmitted by *Culicoides* biting midges. However, in 2008 the first atypical BTV (BTV-25) was identified in goats by metagenomics [6]. Since then, several additional ‘novel’ serotypes have been identified (up to at least BTV-36 [7]) that are characterised by an inability to replicate productively in *Culicoides* cells, effectively preventing transmission by these vectors. These ‘novel’ viruses appear to be primarily transmitted horizontally between ruminants by direct contact between infected and in-contact individuals [8,9,10,11].

Orbiviruses have genomes composed of ten segments of dsRNA (identified as segment 1 (Seg-1) to segment 10 (Seg-10) in order of decreasing size). The orbivirus genome segments encode a total of seven structural viral proteins (VP1-VP7) and at least five non-structural proteins (NS1-NS5) [12,13,14]. Most of the genome-segments encode a single protein from a large open reading frame (ORF) that spans almost the total length of the segment. Seg-9 and Seg-10 also have functional downstream initiation codons within the main ORF, leading to the synthesis of two related proteins in each case (VP6 and VP6a from Seg-9; NS3 and NS3a from Seg-10). Alternate and overlapping ORFs have also been identified in both Seg-9 and Seg-10, encoding non-structural proteins NS4 and NS5, respectively [12,13,14,15].

The genome segments responsible for the blocked replication of BTV-26 in insect cells have been identified using reassortants containing individual BTV-26 genome segments in a BTV-1 genome-backbone. These were engineered by reverse-genetics and used to show that Seg-1 (encoding the viral RNA-dependent RNA polymerase, VP1(Pol)), Seg-2 (encoding the outer capsid and cell attachment protein, VP2(OC1)) and Seg-3 (encoding the inner layer of the core, VP3(T2)) are individually responsible for fully blocking replication in KC cells [16]. Seg-7 of BTV-26 partially blocks the replication of BTV-26 during early stages of infection in *Culicoides* cells and in adult *Culicoides* [16]. None of the three genome segments from BTV-26 (Seg-1, 2 and 3) appear to have significant effects on the replication of BTV-26 or its reassortants in mammalian cells.

The ten BTV mRNAs represent full-length transcripts of the genome segments that are synthesised within the virus cores, in approximately equal amounts by weight (greater numbers of the smaller mRNAs). In vitro studies of transcription frequencies during mRNA synthesis within the core particles of other reoviruses reached similar conclusions. These ratios are not thought to vary significantly under different reaction conditions or cell systems [17,18,19].

Our data suggest that entry of BTV-26 into KC cells is not compromised. Both BTV-1 and BTV-26 bind to the KC cell surface with similar efficiencies. We have therefore further explored the underlying mechanisms attributed to Seg-1, -2 and -3 of BTV-26, that block BTV-26 replication in *Culicoides* cells. The aim of this study is to determine if these mechanisms are linked to a targeted degradation of the viral mRNA derived from Seg-1, -2 or -3 or are due to the characteristics of the proteins translated from them. We present data showing that mRNAs expressed from plasmids containing cDNA copies of BTV-26 Seg-1, 2 and 3 are transcribed at comparable levels in KC cells for both BTV-1 and BTV-26, suggesting that the replication block is less likely due to an RNAi-like response. However, translation of these mRNAs appears to generate BTV-26 proteins that are either poorly expressed or have sizes indicating co-translational or post-translational modifications that may impair their functions, contributing to failure of particle assembly in infected cells.

## 2. Materials and Methods

### 2.1. Cell Lines and Viruses

Baby hamster kidney BSR cells (a clone of BHK-21 cells, [20]) were grown at 37 °C in Dulbecco’s modified Eagle’s medium (DMEM), supplemented with 10% foetal bovine serum (FBS) and 100 IU of penicillin/100 µg of streptomycin per mL, under 5% CO_2_. KC cells which were derived from *Culicoides sonorensis* [21] were grown in ambient air at 28 °C in Schneider’s insect medium supplemented with 10% FBS and 100 IU of penicillin/100 µg of streptomycin per mL.

Wild type BVT-26 (isolate KUW2010/02) was obtained from the dsRNA virus collection at the Pirbright Institute (www.reoviridae.org/dsRNA_virus_proteins/ReoID/BTV-isolates.htm (accessed on 2 November 2021)). BTV-1RG_C7_ is a clone of BTV serotype 1 previously derived by reverse genetics (RG) based on the genome sequence of the BTV-1 reference strain [22]. The genome sequences of BTV-1RG_C7_ and BTV-1 reference strain had multiple nucleotide (and amino acid) differences [22]. The BTV-1RG_C7_ genome backbone was used to create reassortants, by reverse genetics, containing BTV-26 Seg-1 (RGBTV1:1_(26)_), Seg-2 (RGBTV1:2,6,7_(26)_), Seg-3 (RGBTV1:3_(26)_,), Seg-4 (RGBTV1:4_(26)_), Seg-5 (RGBTV1:5_(26)_), Seg-6 (RGBTV1:6_(26)_), Seg-7 (RGBTV1:7_(26)_), Seg-8 (RGBTV1:8_(26)_), Seg-9 (RGBTV1:9_(26)_) or Seg-10 (RGBTV1:10_(26)_). These reassortants are described in our previously published study, investigating which genome segments are involved in direct transmission of BTV-26 between animal hosts [22].

### 2.2. Infection of BSR, C6/36 or KC Cells with BTV-1RG_C7_, BTV-26, RGBTV1:1_(26)_, RGBTV1:2,6,7_(26)_, RGBTV1:3_(26)_, RGBTV1:4_(26)_ or RGBTV1:7_(26)_

Our earlier studies showed that reassortants of the wild-type BTV-1 reference strain, containing BTV-26 Seg-1, -2 or -3, fail to replicate in KC cells [16].

All 10 reassortants in the BTV-1RG_C7_ backbone described in Section 2.1 replicate efficiently in BSR cells [22]. Replication of these reassortants was not previously assessed in KC or C6/36 cells. We therefore assessed replication of wild-type BTV-26, BTV-1RG_C7_ and the novel reassortants in KC or C6/36 cells (MOI of 0.1) grown in 12.5 cm^2^ culture flasks which were seeded with 2 × 10^6^ cells 24 h prior to infection. Infections were carried out in triplicate, and the flasks were incubated at 28 °C. Supernatant volumes of 100 µL were collected daily at regular intervals and stored at 4 °C until the end of the time course on day 14. All aliquots were then titrated by plaque assay on BSR cells as previously described [23]. 

### 2.3. Purification of BTV-1RG_C7_ and BTV-26 Grown in BSR Cells and Assessment of Binding of Virus Particles to Cell Membranes of KC Cells

BTV-1RG_C7_ and BTV-26 were purified from infected BSR cell cultures. Twelve T175 cm^2^ flasks of 90% confluent BSR cells were infected with BTV-1RG_C7_ or BTV-26 at an MOI of 0.1 in DMEM medium containing 2% FBS. Flasks were incubated at 37 °C for 4 days. Cells were harvested by scraping and pelleting at 800× g for 5 min at 4 °C. The supernatant was discarded, and cells were suspended in 10 mL of ice-cold TNE buffer (50 mM Tris/HCI, pH 8.0, 0.2 M NaCl, 5 mM EDTA) containing 0.5% of Triton-X100. Virus purification was performed as previously described [24]. Virus material collected from the final discontinuous gradient was dialysed twice at 4 °C against 3 litres of 0.2 M Tris-HCl pH 8.0 containing 0.1% sodium lauryl sarcosinate. Aliquots of the purified viruses were 10-fold serially diluted in serum-free MEM (Minimal Essential Medium) and titrated by plaque assays as previously described [23]. Purified BTV-1RG_C7_ and BTV-26 particles were titrated in BSR cells and diluted to contain 3.6 × 10^8^ and 1.2 × 10^8^ PFU/mL, respectively.

For binding assays, KC cells were grown on coverslips placed in cell culture plates. Each well was seeded with 10^5^ cells. The cells were washed twice with PBS (calcium/magnesium free) and once with serum-free MEM (Gibco, Thermo Fisher Scientific, Waltham, MA, USA) and then kept on ice. Aliquots of 400 µL of purified virus material, diluted in serum-free MEM, were added per well and incubated on ice for 2 h. Under these conditions KC cells, the MOI of BTV-1 was 140 PFU/cell, and that of BTV-26 was an MOI of 50 PFU/cell.

Cells were washed twice with ice-cold serum-free MEM and once with ice-cold PBS (calcium/magnesium free) followed by fixing with 4% paraformaldehyde solution for 1 h. Mouse anti-VP2 antibodies to BTV-1 or BTV-26, prepared as previously described [23], were diluted with equal volume of PBS and adsorbed by 3 successive incubations (each lasting 30 min) onto monolayers of non-infected KC cells to ensures removal of any mouse antibodies that might cross-react with the cellular proteins. These antibodies were then used to detect BTV-1 or BTV-26 at the surface of KC cells incubated with these viruses, by immunofluorescence microscopy.

For immunofluorescence, the antibodies were diluted 1/500 in PBS containing 0.5% bovine serum albumin (PBS-A) and applied to the fixed cells. After an incubation for 1 h at room temperature, coverslips were washed in PBS and then incubated with Alexa Fluor 488-conjugated anti-mouse IgG (Thermo Fisher Scientific, Waltham, MA, USA) diluted 1/250 in PBS. After labelling with primary and secondary antibodies, the cells were stained with DAPI (1/10,000) for 15 min for nuclear staining and mounted with Vectashield (Vector Laboratories, Newark, CA, USA) for confocal microscopy.

### 2.4. Construction of Expression Plasmids and Assessment of Their Functionality on Insect Cells

Various existing insect expression plasmids were tested in KC cells. The intensity of expressed fluorescent proteins was considered as primary reference to assess their efficacy, and they were inefficient in KC cells.

A Golden Gate destination plasmid designated pCI-G was constructed as described in supplementary data. pCI-G is derived from pCI-neo and allows expression in mammalian cells. Insect polyubiquitin (PUb) promoter drives significantly high levels of expression when used in homologous cell lines [25]. We constructed a mosquito expression plasmid by replacing the CMV promoter of pCI-G with the *Aedes aegypti* PUb promoter, which resulted in the pAE plasmid. Because of the low efficacy of existing insect expression plasmids in KC cells, we constructed a *Culicoides* expression plasmid which we designated pKCi. We therefore identified the nucleotide sequences encoding *Culicoides* PUb using the programme tblastn. Once the nucleotide sequence of the exons had been identified, the region upstream from these exons was analysed using the Neural Network Promoter Prediction software. Putative promoter sequence was PCR amplified from genomic DNA extracted from KC cells using primers shown in Table S1. The PCR product was inserted in pCI-G (described the text provided in supplementary material) to generate plasmid pKCi.

EGFP-6xHis was inserted by Golden Gate cloning into plasmids pAE, pKC and pKCi generating plasmids pAE-EGFP-6xHis, pKC-EGFP-6xHis and pKCi-EGFP-6xHis. These three plasmids were used to transfect C6/36 and KC cells. Transfections were performed using Lipofectamine 2000 (Invitrogen, Carlsbad, CA, USA), Metafectene^®^ Pro (Biontex, München, Germany), Insectogene (Biontex, München, Germany) or TransIT^®^-Insect (Mirus Bio, Madison, Wisconsin, USA) as recommended by the manufacturers, in order to assess the strength of the promoter they carry. Cells were observed by fluorescent microscopy between 18 and 90 h post-transfection, and results are presented in Figures S2 and S3.

### 2.5. Golden Gate Cloning

Expression plasmids compatible with Golden Gate cloning were developed (Figure S4). Plasmids were engineered to contain a SapI restriction site. Golden Gate cloning compatible primers were designed (Table S1) to PCR amplify target ORFs. The cloning reactions contained a destination vector (pCI-G, pAE or pKCi), a pair of Golden Gate entry plasmids, SapI and T4 DNA ligase. The reaction was incubated on a thermal cycler for 1 h and 40 min. The cycling parameters were 1 h at 37 °C, 25 min at 20 °C, 5 min at 50 °C and 10 min at 65 °C. When a target gene contained SapI restriction sites, T4 DNA ligase was further added at 20 °C to ensure the efficient ligation of the full-length target gene.

The entry plasmids are derived from pDONR/Zeo in which multiple cloning sites were introduced into ccdB ORF (Figure S5 and Table S2).

Entry clones containing various ORFs encoding viral proteins and tags (6xHis, Twin-Strep-6xHis-tag or 6xHis-Twin-Strep-tag) were constructed and amplified in DH5α or Stbl2 bacteria, and their sequences were confirmed by Sanger dideoxy sequencing using primers described in Table S1.

### 2.6. Expression of VP1, VP2, VP3 and VP7 of BTV-1RG_C7_ or BTV-26 in BSR or KC Cells Using pCI-G or pKCi

Due to the lack of specific antibodies to most of the proteins which we assessed, short tags were included to help detect protein expression in transfected cells. These include a 6xHis tag and a Twin-Strep-tag.

ORFs of VP1, VP2, VP3 and VP7 of BTV-1 or BTV-26 were used to construct expression plasmids in pKCi or pCI-G with twin tags: 6xHis-Twin-Strep (HS: for tagging at the COOH end) or Twin-Strep-6xHis (SH: for tagging at the NH_2_ end) (Table 1). Primers shown in Table S1 were used to construct the recombinant plasmids which were amplified in DH5α or Stbl2 chemically competent bacteria, and their sequences were confirmed by Sanger dideoxy sequencing.

Cells were grown in 12-well plates. Each well was seeded with 10^6^ cells. Twenty-four hours later, cells were transfected with 1 µg of plasmid per well, using TransIT^®^-Insect as recommended by the manufacturer. Cells were incubated at 28 °C up to 52 h post-transfection (for RNA extractions) or for 4 or 7 days (for Western blot analyses). Cells were scraped and pelleted by centrifugation at 800× *g* for 5 min at 4 °C. Pellets were either dissolved in sample denaturation buffer (160 mM Tris-HCl, 4 mM EDTA, 3.6% SDS, 60 mM DTT, 0.2% ß-mercaptoethanol, 0.8% methionine and 800 mM sucrose) and used for Western blot or used for total RNA extraction with TRizol^®^.

### 2.7. Expression of VP1, VP2, VP3 and VP7 of BTV-1RG_C7_ and/or BTV-26 in C6/36 Cells Using the pAE Plasmid

BTV-26 replicates weakly in C6/36 cells, as compared to wild type BTV-1 [16]. Reassortants RGBTV1:1_(26)_, RGBTV1:2,6,7_(26)_ and RGBTV1:3_(26)_ also replicate in C6/36 cells. RGBTV1:1_(26)_ or RGBTV1:2,6,7_(26)_ showed an initial lag, and virus titres increased to similar levels to those detected with BTV-1RG_C7_, by the end of the time-course experiment on day 14. However, reassortant RGBTV1:3_(26)_ showed a more significant lag up to day 9 in the time-course experiment, then titres increased but remained significantly lower than wild-type BTV-1 [16]. We therefore assessed the expression of VP1, VP2 and VP3 of BTV-26 in C6/36 cells using the pAE expression plasmid. Expression of VP7 was used as a control. Seven expression plasmids were constructed: pAE-BTV1-VP1-HS, pAE-BTV26-VP1-HS, pAE-BTV26-SH-VP2, pAE-BTV1-VP3-HS, pAE-BTV26-VP3-HS, pAE-BTV1-VP7-HS and pAE-BTV26-VP7-HS.

Recombinant plasmids were amplified in DH5α or Stbl2 bacteria, and their sequences were confirmed by Sanger dideoxy sequencing. These plasmids were used to transfect C6/36 cells using ‘TransIT^®^-Insect’, as recommended by the manufacturer. Cells were scraped and pelleted by centrifugation, and the pellet was dissolved in a sample denaturation buffer.

### 2.8. Western Blot

Lysates of KC or C6/36 cells were analysed by SDS-PAGE (10% polyacrylamide gel) and then electro-blotted onto a 0.45 μm nitrocellulose membrane as previously described [23,26]. Membranes were blocked with 20 mL of blocking buffer (PBS 1X, 3% BSA and 0.05% Tween20) at room temperature for 1 h. This was followed by three washes with PBS containing 0.1% Tween20 (PBST). In some experiments, membranes were further blocked by diluting 10 µL of biotin blocking buffer (IBA Lifesciences, Göttingen, Germany) in 10 mL of PBS containing 0.1% Tween20.

Strep-Tactin^®^ HRP (IBA Lifesciences, Göttingen, Germany) was diluted 1/100 in a buffer containing 100 mM Tris-HCl pH 8.8, 100 mM NaCl and 5 mM MgCl_2_. Ten microliters of this preparation were further diluted in 10 mL PBST prior to being added to the membrane, which was incubated for 1 h at room temperature. Membranes were washed twice with PBST for 1 min and then twice with PBS for 1 min at room temperature. Proteins on membranes were revealed by chemiluminescence detection using Clarity™ Western ECL Substrate (Bio-Rad, Marnes-la-Coquette, France). Blots were imaged using the Bio-Rad Chemidoc XRS+.

### 2.9. Quantification of Protein Expression

Relative quantities of recombinant proteins were assessed using Bio-Rad image lab 6.1.0 tools. The software was used to quantify the protein bands of interest by selecting them. The adjusted band volumes, subtracting the background, were calculated by the software. The values calculated in the data analysis table were used to determine the ratios for the desired proteins using a cellular biotinylated protein of approximately 130 kDa.

### 2.10. Design of Real-Time PCR Primers

Real-time PCR primers were designed using the PerlPrimer programme [27]. Several pairs of primers were initially designed and then assessed for their efficiencies and reproducibility of the observed results in real-time PCR. The selected pairs of primers shown in Table S1, were used with the QuantiTect SYBR Green PCR Kit (Qiagen, Les Ulis, France) to quantify mRNA transcribed from expression plasmids in transfected cells.

### 2.11. Assessing Stability of Viral mRNA Derived from Transcription of pKCi Plasmids within KC Cells

Plasmids pKCi-BTV26-VP1-HS, pKCi-BTV26-SH-VP2, pKCi-BTV26-SH-VP3, pKCi-BTV26-VP7-HS, pKCi-BTV1-VP1-HS, pKCi-BTV1-SH-VP2, pKCi-BTV1-SH-VP3 or pKCi-BTV1-VP7-HS were used to transfect KC cells. The experiment was repeated at two separate occasions. In the first experiment, cells were harvested at three time points (6, 24 and 30 h) post-transfection, and during the second experiment an additional time point was added at 52 h. Each plasmid was transfected into two distinct wells per experiment. Prior to harvesting cells, they were washed twice with cold PBS. Cells were scraped and pelleted by centrifugation for 5 min at 700× *g* at 4 °C. Each cell pellet was dissolved in 1 mL of TRIzol reagent, and total RNA purification was performed as directed by the manufacturer. The purified RNA pellets were dissolved in 35 µL of RNAse-free water. Aliquots of 20 µL RNA were treated with 1.5 µL units of turbo DNAse for 90 min at 37 °C followed by further adding 1.5 µL for an additional 90 min. RNA was further purified using RNeasy mini kit (Qiagen, Les Ulis, France), and RNAs were eluted in 40 µL of RNAse-free water. Prior to reverse transcription, 2 µL of random hexa-primers (Roche, Basel, Switzerland) was added to 10 µL of RNA, and the mixture was heat-denatured at 72 °C for 6 min. Twenty-seven units of AMV reverse transcriptase (Roche, Basel, Switzerland) were added, and the reaction was incubated at 37 °C for 75 min. Real-time PCR was performed in triplicate. Each reaction mixture contained 2 µL of cDNA, 0.5 µM of the final concentration of each primer and 6.25 µL of 2x QuantiTect master-mix, with a final volume of 12.5 µL. PCR cycling parameters were as follows: one cycle at 95 °C for 15 min, followed by 40 cycles of 94 °C for 15 sec, 52 °C for 25 sec and 72 °C for 20 sec. A melt curve analysis was performed at the end of cycling in order to assess the dissociation characteristics of the PCR product and confirm the existence of a single amplicon.

### 2.12. Bioinformatic Analyses

Codon usage and the existence of potential rare codons were analysed using the codon usage calculator from the Sequence Manipulation Suite (https://www.bioinformatics.org/sms2/codon_usage.html (accessed on 6 March 2023)) [28], using 21,241 *Culicoides sonorensis* annotated CDS from VectorBase (https://vectorbase.org/common/downloads/release-61/CsonorensisPIR-s-3/fasta/data/VectorBase-61_CsonorensisPIR-s-3_AnnotatedCDSs.fasta (accessed on 6 March 2023)). The GC content and identification of GC-rich regions in the viral nucleic acid sequences were analysed using a sliding window of 300 nucleotides and the GC content calculator (https://www.biologicscorp.com/tools/GCContent (accessed on 6 March 2023)). RNA secondary structures and minimum free energy (MFE) were inferred using the ‘RNAfold’ programme, implemented in the ‘ViennaRNA’ package (version 2.0) [29]. Models of protein tertiary structures were investigated using Phyre2 (http://www.sbg.bio.ic.ac.uk/phyre2 (accessed on 27 January 2023)), and structures were visualised using ChimeraX software (version 1.5) [30]. Prediction of post-translational modifications in proteins was performed using musite (https://www.musite.net (accessed on 27 January 2023)).

## 3. Results

### 3.1. Infection of BSR, C6/36 or KC Cells with BTV-1RG_C7_, BTV-26, RGBTV1:1_(26)_, RGBTV1:2,6,7_(26)_, RGBTV1:3_(26)_, RGBTV1:4_(26)_ or RGBTV1:7_(26)_

**Figure 1:**
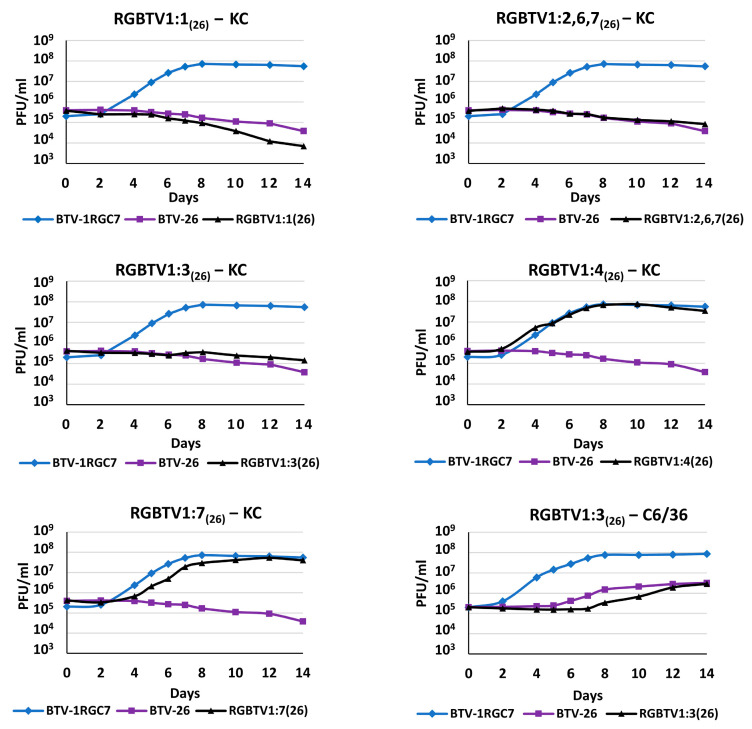
Replication of reassortants of BTV-26 in the BTV-1RG_C7_ backbone in *Culicoides sonorensis* KC or *Aedes albopictus* C6/36 cells. The curves show virus titres in PFU/mL of cell culture supernatant over a time course of 14 days. Each virus was tested in three separate time course experiments, and titres were determined by a plaques assay, in BSR cells as described in materials and methods.

BTV-1RG_C7_ and BTV-26 replication in the BSR, C6/36 and KC cells was assessed by plaque assay. For BTV-1RG_C7_, titres in the three cell lines at the plateau phase were ~3 × 10^8^, ~5.5 × 10^7^ and ~8 × 10^7^ PFU/mL of cell lysates, respectively. In BSR cells, the virus titre reached by BTV-26 was ~2 × 10^8^, which is comparable the titre of BTV-1RG_C7_ in the same cells. However, titres of BTV-26 at the plateau phase in C6/36 cells were ~2.8 × 10^6^ PFU/mL, which represents 1.29 log_10_ or 95% less virus than BTV-1RG_C7_ in the same cells. Finally, BTV-26 fails to replicate in KC cells (Figure 1).

Replication of reassortants RGBTV1:1_(26)_, RGBTV1:2,6,7_(26)_ and RGBTV1:3_(26)_ is blocked in KC cells (Figure 1) [11,12]; however, the titres of RGBTV1:7_(26_) generated by the end of the time course were not significantly different from those of BTV-1RG_C7_ (Figure 1). RGBTV1:4_(26)_ was used as a control, replicating at similar titres to BTV-1RG_C7_.

### 3.2. Binding of Purified Virus Particles of BTV-1RG_C7_ and Wild-Type BTV-26 to Cell Membranes of KC Cells

**Figure 2:**
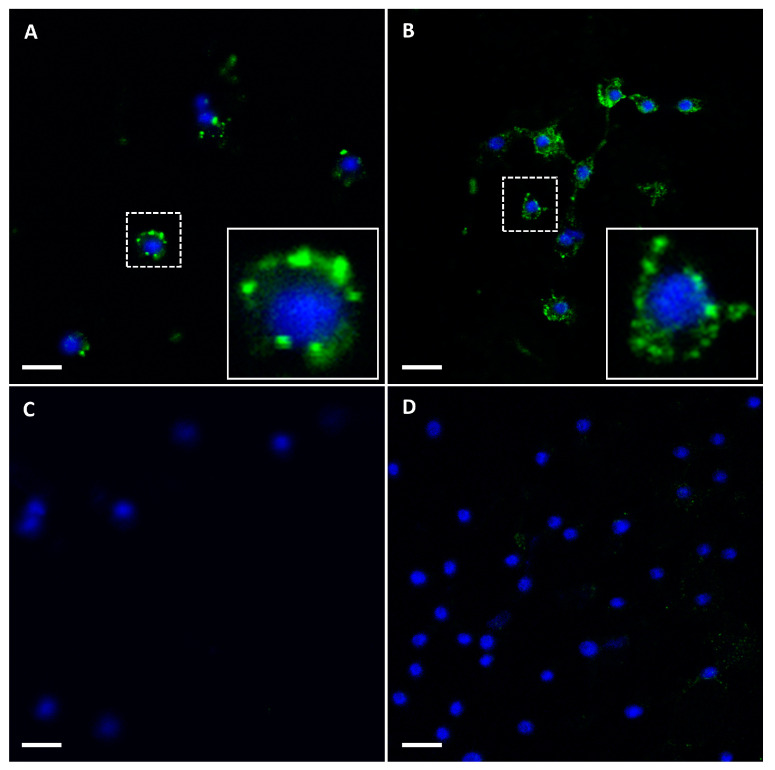
Binding of BTV particles to the KC cell surface. Gradient purified BTV-1RG_C7_ and BTV-26 were diluted in MEM and bound to monolayers of KC cells at 0 °C. The presence of virus particles bound to cell surface was detected by confocal immunofluorescence using mouse antibodies to recombinant VP2s of BTV-1 or BTV-26 and Alexa Fluor 488-conjugated anti-mouse IgG. (**A**) KC cells incubated with BTV-1RG_C7_ showing green fluorescence as identified by anti-VP2 of BTV-1 antibodies. (**B**) KC cells incubated with BTV-26 showing green fluorescence as identified by anti-VP2 of BTV-26 antibodies. (**C**,**D**) KC cells, in the absence of purified virus particles, probed with anti-VP2 of BTV-1 (**C**) or anti-VP2 of BTV-26, respectively (**D**). Nuclei were stained with DAPI (blue). In the lower right corner of panels (**A**,**B**), a single (cell indicated by a box with a dotted white line) is shown enlarged (indicated by a box with a solid white with line) with green fluorescence decorating the cell surface, indicating binding of the virus. Scale bar represents 5 µm.

KC cells were incubated with either BTV-1 (MOI of 140 PFU/cell) or BTV-26 (MOI of 50 PFU/cell). Interaction of the viruses with the cell surface was analysed by confocal immunofluorescence microscopy, which showed that both viruses bound to the cell surface efficiently (Figure 2).

### 3.3. Assessing Stability of Viral mRNA Derived from Transcription of pKCi Plasmids within KC Cells

**Figure 3:**
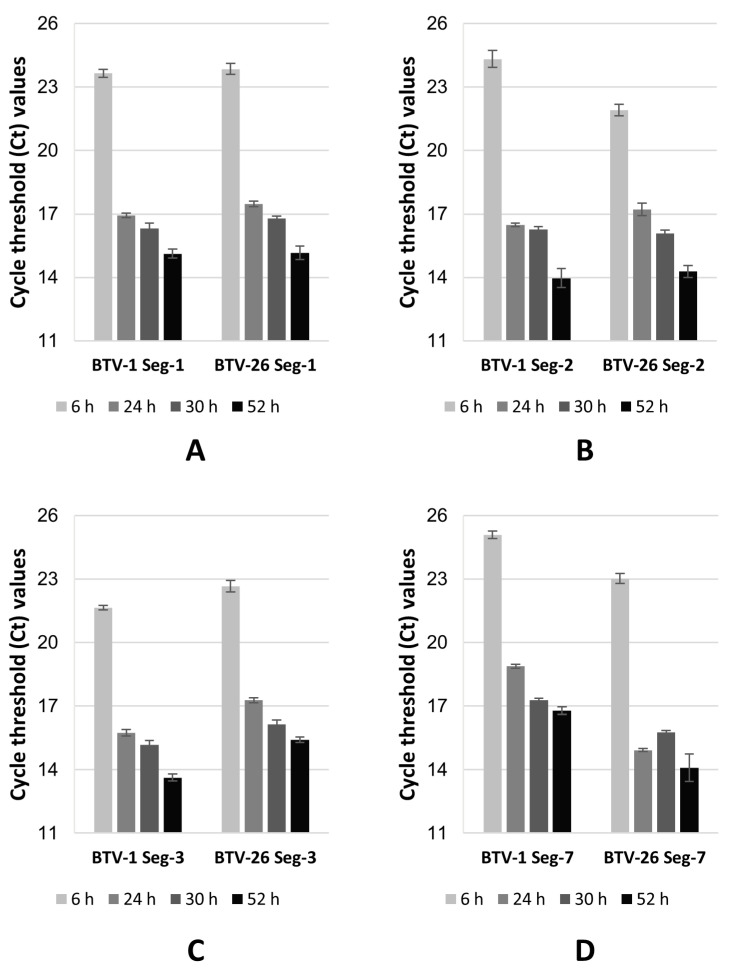
Real-time PCR Ct values for the mRNAs transcribed from plasmids in KC cells. Plasmids pKCi-BTV1-VP1-HS and pKCi-BTV26-VP1-HS (**A**), pKCi-BTV1-SH-VP2 and pKCi-BTV26-SH-VP2 (**B**), pKCi-BTV1-SH-VP3 and pKCi-BTV26-SH-VP3 (**C**) and pKCi-BTV1-VP7-HS and pKCi-BTV26-VP7-HS (**D**) were transfected into KC cells. The RNA extracts were reverse transcribed using hexanucleotide primers followed by PCR using primers described in Table S1. The results indicate that the levels of mRNAs increased over the time course between 6 h and 52 h post-transfection.

The mRNAs from Seg-1, Seg-2, Seg-3 and Seg-7 of BTV-1 or BTV-26 were extracted from KC cells harvested at 6, 24, 30 and/or 52 h post-transfection with the corresponding pKCi plasmid. Real-time PCRs using segment-specific and serotype-specific real-time PCR primers (see Table S1) showed that levels of mRNAs increased (Ct values reduced) over a 52 h time course (Figure 3 and Figure S6). These results (observed in two separate experiments) indicate that the viral mRNAs expressed using the pKCi plasmids are not rapidly degraded by cellular nucleases.

In the case of Seg-1, the transcription levels of derived mRNAs from plasmids pKCi-BTV1-VP1-HS and pKCi-BTV26-VP1-HS, used to transfect KC cells, were comparable. The amplification efficiencies calculated for the two sets of primers (Table S3) used for real-time PCR were also relatively similar. The ΔCt between the initial and end time-points is comparable for both mRNAs (BTV-1 = 8.53 and BTV-26 = 8.69).

For Seg-2, the transcription levels of mRNAs derived from plasmids pKCi-BTV1-SH-VP2 and pKCi-BTV26-SH-VP2 were higher (2.4 Ct lower) when tested at 6 h for KC cells transfected with the latter plasmid. The amplification efficiencies calculated for the two sets of primers (Table S3) used for real-time PCR were relatively similar. Overall, the ΔCt between the initial (6 h) and end (52 h) time-points is higher in case of BTV-1 mRNA (BTV-1 = 10.35 Ct as compared to BTV-26 = 7.64 Ct), which indicates about six-fold more mRNA was accumulated from transcription of pKCi-BTV1-SH-VP2 between 6 and 52 h, although the Ct values at 24, 30 and 52 h are similar for BTV-1 and BTV-26 mRNAs.

Up to 1.5 Ct higher values (lower mRNA levels) were consistently observed for mRNAs transcribed in KC cells transfected with the pKCi-BTV26-SH-VP3 plasmid, as compared to pKCi-BTV1-SH-VP3, even though the efficiencies calculated for different primers sets (Table S3) indicate a higher efficiency for primers derived from Seg-3 of BTV-26. The ΔCt between the initial (6 h) and end (52 h) time-points is comparable for both mRNAs (BTV-1 = 8.01 Ct and BTV-26 = 7.25 Ct).

Transcription levels of Seg-7 derived mRNAs of BTV-1 (plasmid pKCi-BTV1-VP7-HS were lower than those calculated for BTV-26 (pKCi-BTV26-VP7-HS) even though the efficiencies calculated for the two sets of primers (Table S3) used for real-time PCR were relatively similar. The ΔCt between the initial (6 h) and end (52 h) time-points is comparable for both mRNAs (BTV-1 = 8.3 Ct and BTV-26 = 8.92 Ct).

Given the steady accumulation of mRNAs in KC cells transfected with the plasmids, it is unlikely that an RNAi response targeting mRNAs derived from genome Seg-1, -2 or -3 block replication of BTV-26 in insect cells.

### 3.4. Expression of VP1, VP2, VP3 and VP7 of BTV-1RG_C7_ or BTV-26 in BSR Cells Using the pCI-G Plasmid

**Figure 4:**
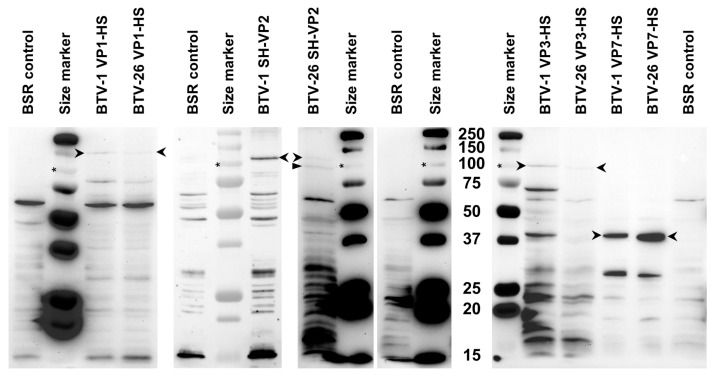
Expression of VP1, 2, 3 and 7 or BTV-1 or BTV-26 in BSR cells. All proteins are expressed as full-length proteins (indicated by open arrowheads). Note that VP2 of BTV-26 expresses as a full-length protein but also a truncated form that is ~10 kDa shorter (indicated by a closed arrowhead). These results were reproducible at two separate occasions. Asterisk indicates the position of the 100 kDa size marker.

BSR cells transfected with pCI-G plasmids expressing VP1, VP2, VP3 and VP7 of BTV-1 or BTV-26 were harvested 48 h post-transfection and analysed by SDS-PAGE and Western blot using Strep-Tactin-HRP (IBA Lifesciences, Göttingen, Germany). All four proteins of BTV-1 had sizes (Figure 4) corresponding to their theoretical molecular weights (Table 2) calculated from the open reading frames. VP1, VP3 and VP7 of BTV-26 also had sizes (Figure 4) corresponding to their theoretical molecular weights (Table 2). However, NH_2_-terminal tagged VP2 of BTV-26 was expressed as two major bands, one corresponding to the full-length protein and a second ~10 kDa shorter band (ratio 1:5) (Figure 4).

### 3.5. Expression of VP1, VP2, VP3 and VP7 of BTV-1RG_C7_ and/or BTV-26 in C6/36 Cells Using pAE Plasmids

Our study showed that BTV-26 replicates to low levels in C6/36 cells (1.6 log_10_ or 96% lower than BTV-1RG_C7_, Figure 1) [16]. C6/36 cells transfected with pAE plasmids (pAE-BTV1-VP1-HS, pAE-BTV1-VP3-HS, pAE-BTV1-VP7-HS) COOH-terminal tagged VP1, VP3 and VP7 of BTV-1, harvested at 72 h post-transfection, were analysed by SDS-PAGE and Western blot using Strep-Tactin-HRP.

**Figure 5:**
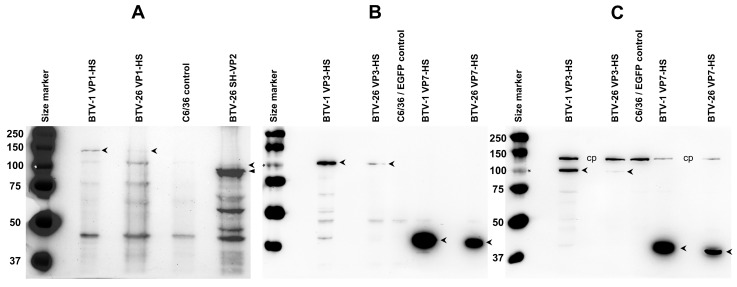
Expression of NH_2_-terminal tagged (SH) or COOH-terminal tagged (HS) VP1, 2, 3 and 7 of BTV-1 or BTV-26 in C6/36 cells transfected with plasmids pAE-BTV1-VP1-HS, pAE-BTV26-VP1-HS, pAE-BTV26-SH-VP2, pAE-BTV1-VP3-HS, pAE-BTV26-VP3-HS, pAE-BTV1-VP7-HS and pAE-BTV26-VP7-HS. These proteins were expressed as full-length products (indicated by open arrowheads) and analysed by SDS PAGE. In panels (**A**,**B**), the blots were performed in the presence of biotin blocking buffer. (**A**) Lower levels of BTV-26 VP1 were observed despite loading 2.5 times more than the amount of total proteins, as compared to VP1 of BTV-1. VP2 of BTV-26 is expressed as full-length protein (indicated by open arrowhead) at low levels and significantly higher levels of a truncated form that is ~10 kDa shorter (indicated by a closed arrowhead). (**B**) Expression of VP3 of both BTV-1 and BTV-26 was observed as the full-length protein (indicated by open arrowhead), although that of BTV-26 was poorly expressed. VP7 of both BTV-1 and BTV-26 were expressed as full-length products (indicated by open arrowhead). (**C**) The same products loaded in panel (**B**), in the absence of biotin blocking buffer, showing the intracellular biotinylated protein (indicated as cp) which was used as a loading control. Asterisk indicates the position of the 100 kDa size marker band.

VP1 of BTV-1 (plasmid pAE-BTV1-VP1-HS) was expressed as a full-length protein in transfected C6/36 cells with a size corresponding to its theoretical molecular weight (Figure 5A). By contrast, lower levels of BTV-26 VP1 (plasmid pAE-BTV26-VP1-HS) were observed, despite loading 2.5 times more the amount of lysate. Our previous studies [16] indicated that the reassortant of genome Seg-1 of BTV-26 in the wild-type BTV-1 reference strain backbone replicates in C6/36 cells with an initial lag but reaches virus titres similar to those of wild-type BTV-1 by two weeks post-infection, possibly reflecting lower levels of synthesis of the VP1 polymerase.

NH_2_-terminal tagged VP2 of BTV-26 was mainly expressed in C6/36 cells (transfected with pAE-BTV26-SH-VP2 plasmid) as the shorter form identified in BSR cells transfected with pCI-BTV26-SH-VP2, although lower levels of the full-length VP2 were also detected in the C6/36 cells (ratio 1:13) (Figure 5A). The reassortant of genome Seg-2 of BTV-26 in the wild-type BTV-1 reference strain backbone [16] also replicates with an initial lag, but again reaches titres similar to wild-type BTV-1 by two weeks post-infection.

VP3 of BTV-26 was poorly expressed (5% of the amount of BTV-1 VP3) in C6/36 cells transfected with pAE-BTV26-VP3-HS (Figure 5B,C), although its estimated size corresponds to its theoretical molecular weight. Our reassortant RGBTV1:3_(26)_ replicates with a much-pronounced lag in C6/36 cells, although viral titres start to increase modestly by day 8 post-infection, reaching similar titres to BTV-26 by day 14, but these remain 1.6 log_10_ (or 96%) lower than titres reached by BTV-1RG_C7_ (Figure 1). These results suggest that the VP3 protein of BTV-26 represents the major factor which influences the final titre reached by BTV-26 in C6/36 cells at day 14 post-infection.

No differences were observed between the levels of expression of VP7 of BTV-1 and BTV-26 in C6/36 cells (Figure 5B,C). This is compatible with previous findings [16], showing that the reassortant of Seg-7 of BTV-26 in the wild-type BTV-1 reference strain backbone replicates with similar efficiency as wild-type BTV-1 in C6/36 cells.

### 3.6. Expression of VP1, VP2, VP3 and VP7 of BTV-1RG_C7_ or BTV-26 in KC Cells Using pKCi Plasmids

KC cells transfected with pKCi plasmids (Table 1) expressing NH_2_-terminal or COOH-terminal tagged VP1, VP2, VP3 and VP7 of BTV-1 or BTV-26 were harvested 4 days post-transfection and analysed by SDS-PAGE and Western blot.

#### 3.6.1. Expression of VP1

**Figure 6:**
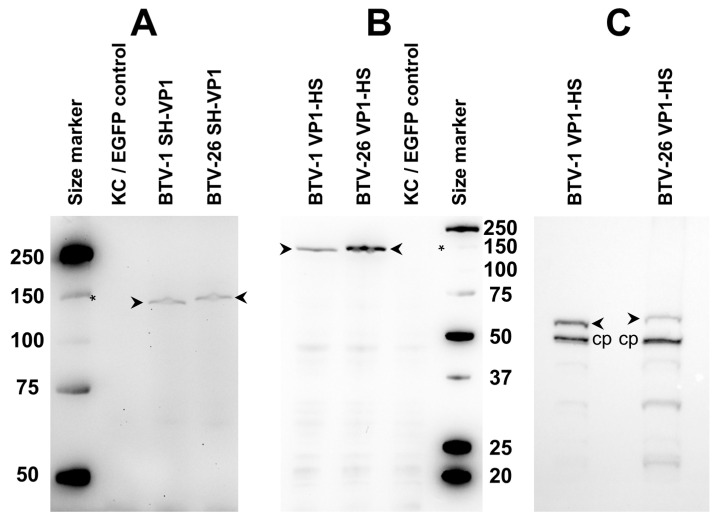
Expression of VP1 of BTV-1 or BTV-26 in KC cells. VP1 proteins of both BTV-1 and BTV-26 were expressed as full-length products in KC cells transfected with pKCi plasmids (pKCi-BTV26-VP1-HS, pKCi-BTV26-SH-VP1, pKCi-BTV1-VP1-HS and pKCi-BTV1-SH-VP1). Panel (**A**) NH_2_-terminal tagged VP1 (indicated by open arrowhead), panels (**B**,**C**): COOH-terminal tagged VP1 (indicated by open arrowhead). (**A**,**B**) Proteins detected using Strep-Tactin-HRP in the presence of biotin blocking buffer. (**C**) Proteins detected with Strep-Tactin-HRP without biotin blocking buffer. This panel also shows the intracellular biotinylated protein (indicated as cp) which was used as a loading control. Systematically, these Western blots show that VP1 of BTV-26, expressed in KC cells, exhibits a molecular weight that is slightly higher than that of the VP1 of BTV-1. The asterisk indicates the position of the 150 kDa size marker band.

The NH_2_-terminal or COOH-terminal tagged VP1 of BTV-1 or BTV-26 in cells transfected with pKCi plasmids was expressed as full-length products (Figure 6 and Figure S7). However, a 10-times longer exposure time was needed to detect the NH_2_-terminal tagged VP1s (Figure S7A). Western blot analysis performed in the absence of the biotin blocking buffer to allow using the internal biotinylated cellular protein as a loading control indicated that the expression levels of the COOH-terminal tagged VP1 from BTV-26 were approximately only one third of those detected for VP1 of BTV-1 (Figure 6C). Regardless of the conditions used for Western blots or the position of the tag, the VP1 of BTV-26 migrated to a position suggesting that it is 6–7 kDa larger than the VP1 of BTV-1, (which is 6–7 kDa higher than the theoretical molecular weight of VP1 of BTV-1 or BTV-26, Table 3).

The results in KC cells contrast with the expression of VP1 in C6/36 cells (in which BTV-26 replicates, at low levels) where both VP1 of BTV-1 and BTV-26 appear to exhibit nearly identical sizes. This apparent difference of 6–7 kDa in the molecular weight between the two VP1s was not observed when the proteins were expressed in BSR cells (in which BTV-26 replicates efficiently), suggesting that the VP1 of BTV-26 is subjected to some form of post-translational modification during expression in KC cells.

**Figure 7:**
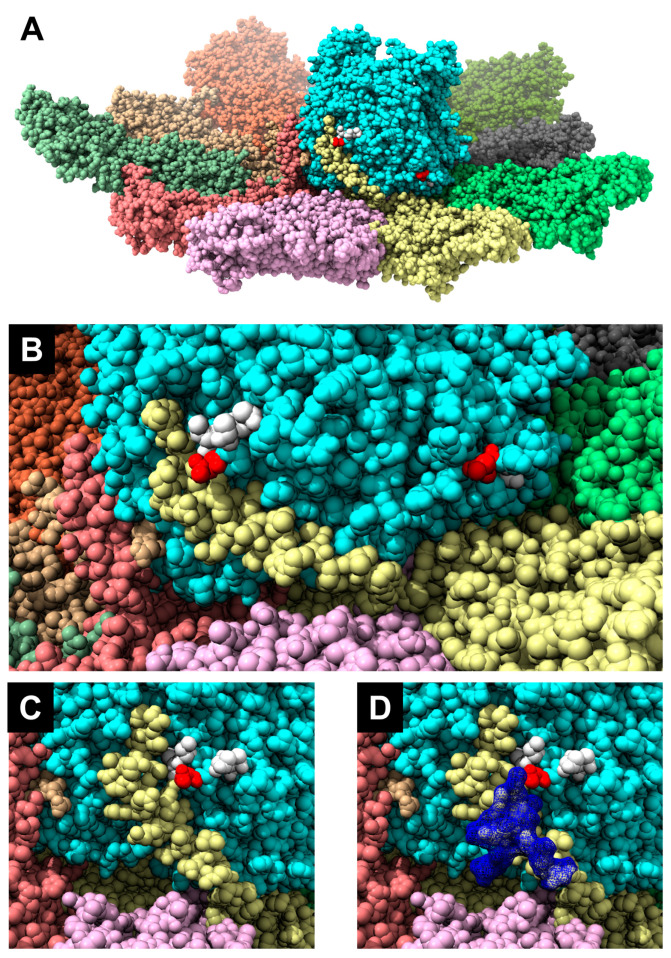
Glycosylation sites specific to BTV-26 VP1. The structure of the VP1/VP3 decamer complex, previously determined at a resolution of 3.60 Å [34], was used to display the two glycosylation sites (positions 226–228 and 777–779) specific for VP1 of BTV-26 with the help of the Chimera programme. The sites and surrounding environment were mutated so as to display the BTV-26 sequences NIT at position 226–228 and NKT at position 777–779. (**A**) view of the whole VP1/VP3 complex showing both theoretical glycosylation sites exposed on the cyan-coloured VP1 surface (the asparagine residues are displayed in red). (**B**) A zoom into the region of panel (**A**) which contains the Asparagine residues that are part of the theoretical glycosylation sites. Apart from the asparagine residues, the remaining mutated sites in the VP1 amino acid sequence are displayed as white spheres. The NH_2_-terminal part of the VP3 chain J in the decamer, displayed in yellow, lies in close proximity to the NIT glycosylation site of VP1. (**C**,**D**) orthogonal views of the VP1/VP3 surface, focused on the NIT glycosylation site, with (**D**) showing the steric hindrance which may result from the volume occupied by a glycan chain of 8 sugar moieties (blue). The composition and nomenclature of the N-Glycan is shown in Figure S9.

The VP1 proteins of BTV1 and BTV-26 were checked for predicted N-glycosylation sites. Two N-glycosylation sites specific to the VP1 of BTV-26 were predicted with a high probability score (>0.904), located at positions 226–228 (NIT) and 777–779 (NKT). These two sites are exposed on the surface of the polymerase structure (Figure 7). Glycosylation in insect cells would add short chains of sugars [31,32,33] with a size of up to 3 kDa per site (up to 15 sugar moieties per site), thus potentially increasing the VP1 size by approximately 6 kDa. The site at 777–779 is only 11 amino acids downstream from the GDD motif (in the nucleotide triphosphate entry channel) of the polymerase, located close to the basal part on the outer surface of the polymerase (Figure 7). The NIT site is juxtaposing the NH_2_ end of a VP3 monomer (J chain in PDB entry 6PO2) (Figure 7 and Figure S8).

The amino acids surrounding the NIT site (EE**NIT**TLE) are fully conserved in at least some of the other atypical BTV serotypes including BTV-25, BTV-28, BTV-X-ITL2015 and ITL2021 (serotype 32). Figure 7 (panel D) shows the steric hindrance which a glycosyl chain of eight sugar moieties (Figure S9) could engender thus possibly interfering with the anchoring of the amino terminal end of a VP3 subunit into a grove located at the lower outer surface of the VP1 and therefore potentially influencing both BTV particle assembly and replication.

#### 3.6.2. Expression of VP2 and Analysis of the RNA Sequence Context

**Figure 8:**
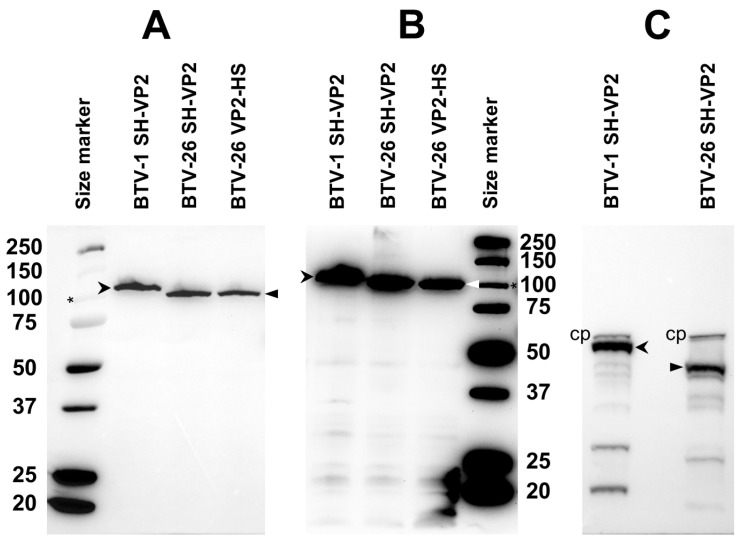
Expression of VP2 of BTV-1 and BTV-26 in KC cells. NH_2_-terminal tagged (SH) VP2 of BTV-1 was expressed in KC cells transfected with pKCi plasmids (pKCi-BTV1-SH-VP2, pKCi-BTV26-SH-VP2 or pKCi-BTV26-VP2-HS). An expressed NH_2_-terminal tagged VP2 of BTV-1 was detected (indicated by open arrowheads), migrating at the appropriate position for the full-length protein (~116 kDa). NH_2_-terminal tagged (SH) and COOH-terminal tagged (HS) VP2 of BTV-26 (indicated by closed arrowheads) migrated at a size that is ~10 kDa smaller than the theoretical molecular weight of VP2. Panels (**A**,**B**) show two different preparations of the VP2s with different exposures, and both panels indicate that VP2 of BTV-26 has a molecular weight that is close to 100 kDa. (**C**) Proteins detected with Strep-Tactin-HRP without biotin blocking buffer showing the intracellular biotinylated protein (indicated as cp) used as the loading control. The asterisk indicates the position of the 100 kDa size marker band.

NH_2_-terminal tagged VP2 of BTV-1 was expressed in KC cells transfected with pKCi-BTV1-SH-VP2 plasmid. Analysis by SDS-PAGE identified the expressed protein at ~116 kDa, corresponding to the theoretical size of the tagged VP2. In contrast, expression of the NH_2_-terminal tagged VP2 of BTV-26 generated a protein that migrated at ~103 kDa, which is ~10 kDa shorter than the protein’s theoretical size, consistent with the shorter of the two forms of BTV-26 VP2 identified in both BSR and C6/36 cells (Figure 4 and Figure 5). The full-length protein was not detected in KC cells, despite multiple expression attempts. We also expressed COOH-terminal tagged VP2 of BTV-26 in KC cells and analysed the expressed protein by SDS-PAGE and Western blotting. The resulting protein also appeared to be ~10 kDa shorter than the theoretical size for the full-length VP2 and has an identical size to the NH_2_-terminal tagged VP2 (Figure 8). The detection of both tagged versions of this protein indicates that it had not been truncated at either terminus. This raises questions about the mechanism that could lead to expression of a short form of VP2.

**Figure 9:**
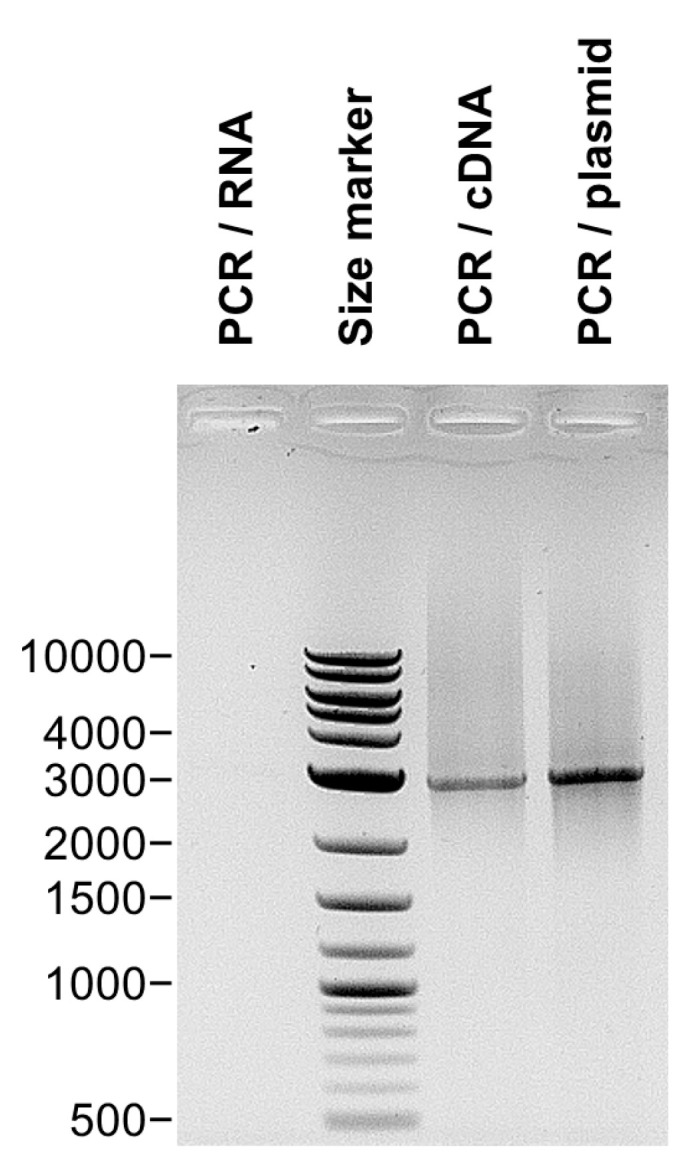
PCR amplification of the BTV-26 VP2 gene. PCR amplification was performed with primers MB324 and MB325 (Table S1) on: DNAseI-treated RNA extracts of KC cells transfected with pKCi BTV-26 VP2-HS; cDNA from AMV reverse transcribed DNAseI-treated RNA extracts of KC cells transfected with pKCi BTV-26 VP2-HS; plasmid pKCi BTV-26 VP2-HS used to transfect KC cells. The PCR products were analysed by electrophoresis in a 1% agarose gel. The expected full-length size of the amplicon is approximately 2900 bp. The results indicate no size differences between the cDNA generated from the VP2 mRNA and the DNA copy cloned in the pKCi plasmid, excluding therefore the possibility of a spliced VP2 mRNA form. No amplified product was detected directly from the extracted RNA. The size marker is labelled in base pairs.

Truncation of the VP2 of an orbivirus has been shown for an isolate of AHSV-7. This VP2 has lost 225 aa (~26 kDa) [35]. Sequencing of the genome segment encoding this truncated AHSV VP2 showed a shorter nucleotide sequence with an internal deletion that did not alter the reading frame. Therefore, we tested whether the mRNA encoding the BTV-26 VP2 has been truncated by a similar mechanism. Total RNA (treated with DNAse I) was extracted from KC cells transfected with pKCi-BTV26-VP2-HS and then reverse transcribed using primer MB426 (Table S1) located at the 3’ end of the mRNA encoding the VP2. PCR was performed on the extracted RNA, cDNA and the pKCi-BTV26-VP2-HS plasmids with primers encompassing the whole coding sequence of VP2 (primers MB324 and MB325, Table S1) and analysed by agarose gel electrophoresis (Figure 9). The products from the cDNA and plasmid DNA generated similar PCR products, indicating that the apparently shorter size of the BTV-26 VP2 does not result from a truncation of the mRNA (e.g., by cleavage or splicing) either during or post-transcription. No amplified products were detected by PCR performed directly on the extracted RNA.

The possibility that the shorter form of the BTV-26 VP2 is due to a co-translational event that occurs with varying frequencies in different cells and/or hosts still needs to be explored. Possible explanations include ribosome jump/shunt/bypass of highly stable mRNA secondary structures.

**Figure 10:**
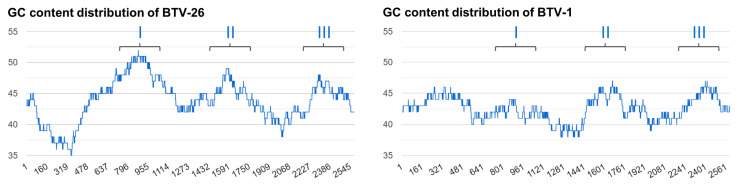
GC content of BTV-1 and BTV-26 Seg-2 ORF nucleotide sequence using a sliding window of 300 nucleotides. The GC calculator programme (see Section 2.12) indicates the presence of three GC rich regions in the sequence of BTV-26 Seg-2 (as compared to equivalent regions in BTV-1). In particular, BTV-26 region I has a high GC content over a stretch between nucleotides 500 and 1200.

We investigated the presence of stable stem-loop structures that would be compatible with the expression of a ~10 kDa shorter BTV-26 VP2, within the sequence of Seg-2 of BTV-26. The GC content calculator identified three structured regions in the sequence of the ORF of BTV-26 Seg-2, with region I having a higher GC content (up to 8% higher) than the other classical BTV serotypes which are transmitted by *Culicoides* vectors (Table 4, Figure 10), although the mean GC content for the entire Seg-2 is comparable between the vectored BTV strains and BTV-26 (42.4 and 44%, respectively).

**Figure 11:**
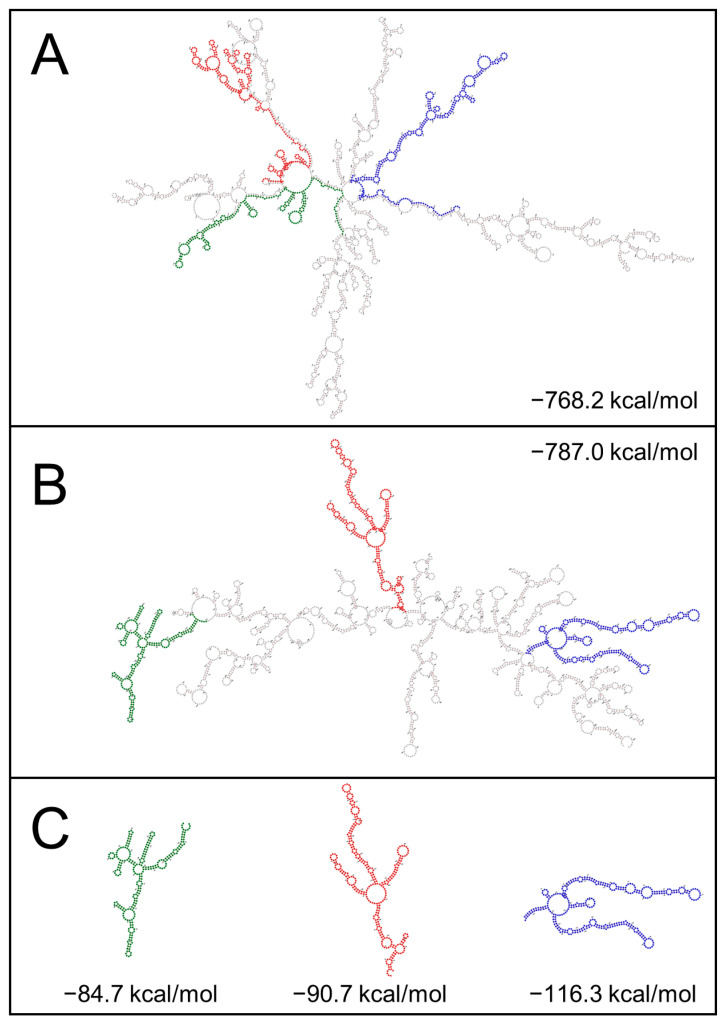
Secondary structures for the full-length ORFs of Seg-2 mRNAs. Secondary structures of full-length ORFs of Seg-2 mRNA from BTV-1 (**A**) or BTV-26 (**B**) generated by RNAFold. The three GC rich regions were identified in the sequence of BTV-26 by GC calculator (using a 300 nt sliding window) are coloured in green, red and blue, respectively. The three homologous regions of BTV-1 mRNA do not form full end-to-end stem-loop structures and do not exhibit a high GC content, as shown in Figure 10. (**C**) Secondary structure folds for individual sequences of regions I, II and III of BTV-26 Seg-2. The secondary structures are identical whether the fold is performed using the full-length sequence of the BTV-26 Seg-2 mRNA or the three individual sequences of these regions.

Secondary structures prediction for the RNA sequences of these three regions of Seg-2 of BTV-1, -4, -6, -9 or -26 suggested that the sequences derived from BTV-26 form the most stable structures, as indicated by the calculated MFE (minimum free energy) values (Table 4). The predicted fold of region I, II or III of Seg-2, from BTV-1, -4, -6, -9 or -26, identified stem loop structures that are conserved, irrespective of whether they are predicted from sequences of full-length mRNA ORFs, or for the individual regions separately (Figure 11). If a ribosome jump occurred in any of these three regions of the Seg-2 mRNA, it would cause the VP2 of BTV-26 to be 10–13 kDa shorter, compatible with the size estimates from SDS-PAGE gels (Table 3).

Attempts were made to identify elements that might be located upstream or at the start of any of the three stem-loops regions of the Seg-2 mRNA, which might cause ribosomes to pause/slow-down. None of the rare codons used by *Culicoides* (in 21,241 annotated *C. sonorensis* CDS) (Table S4)) were detected within the context of the stem-loops using a codon-usage calculator. Codon usage was comparable for full-length ORFs of genome Seg-2 from different BTV serotypes which we analysed (BTV-1, -4, -6, -9 and -26) (Table S4).

#### 3.6.3. Expression of VP3

**Figure 12:**
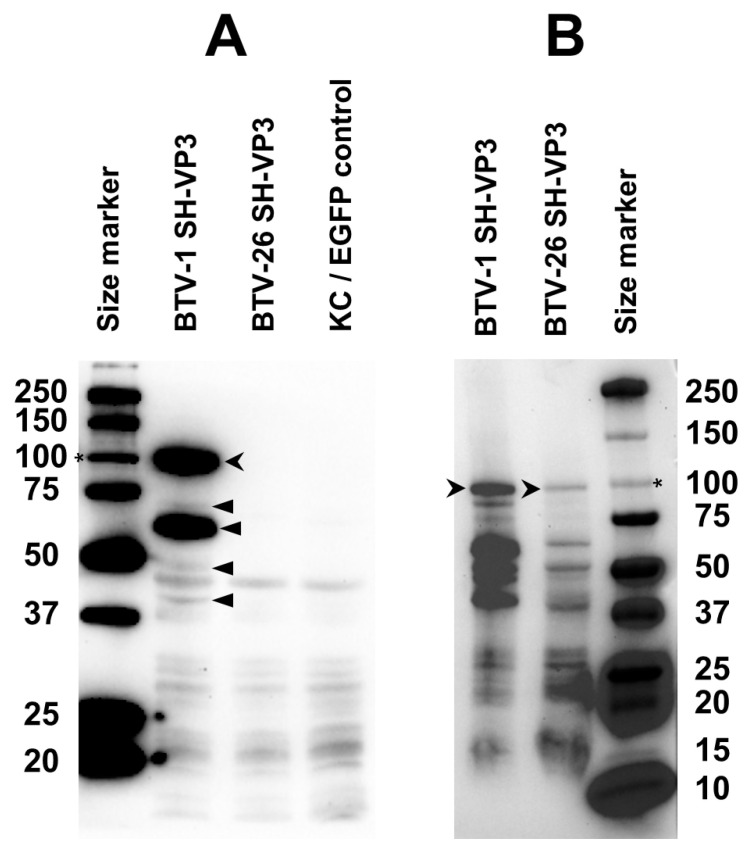
Expression of NH_2_-terminal tagged VP3 of BTV-1 and BTV-26 in KC cells transfected with pKCi-BTV26-SH-VP3 or pKCi-BTV1-SH-VP3. KC cells were harvested at 4 or 7 days post-transfection. (**A**) At 4 days, VP3 of BTV-1 (indicated by open arrowheads) was expressed as a full-length product with a size compatible to its theoretical molecular weight. Specific degradation products of VP3 of BTV-1 are indicated by closed arrowheads. The VP3 of BTV-26 was not detected in cells harvested at 4 days. (**B**) In cells harvested at 7 days, VP3 of BTV-1 or BTV-26 (indicated by open arrowheads) was both detected, but with lower levels for the VP3 of BTV-26. Asterisk indicates the position of the 100 kDa size marker band.

Expression of VP3 of BTV-1 or BTV-26 tagged at the NH_2_ terminal was assessed following transfection of KC cells with plasmids pKCi-BTV1-SH-VP3 or pKCi-BTV26-SH-VP3. At 4 days post-transfection we observed expression of a protein with an apparent molecular weight of ~100 kDa for BTV-1 (corresponding to the theoretical size of the VP3) but did not observe any detectable expression of BTV-26 VP3 (Figure 12A and Figure S10A). The detection of shorter peptides during SDS-PAGE analysis suggests that VP3 appears to be degraded in KC cells (Figure 12 and Figure S10A). VP3 of BTV-26 appears to be more sensitive to degradation and accumulates slowly but steadily and is detected in cells harvested at 7 days post-transfection, though at lower levels as compared with the VP3 of BTV-1 (Figure 12B and Figure S10B).

#### 3.6.4. Expression of VP7

**Figure 13:**
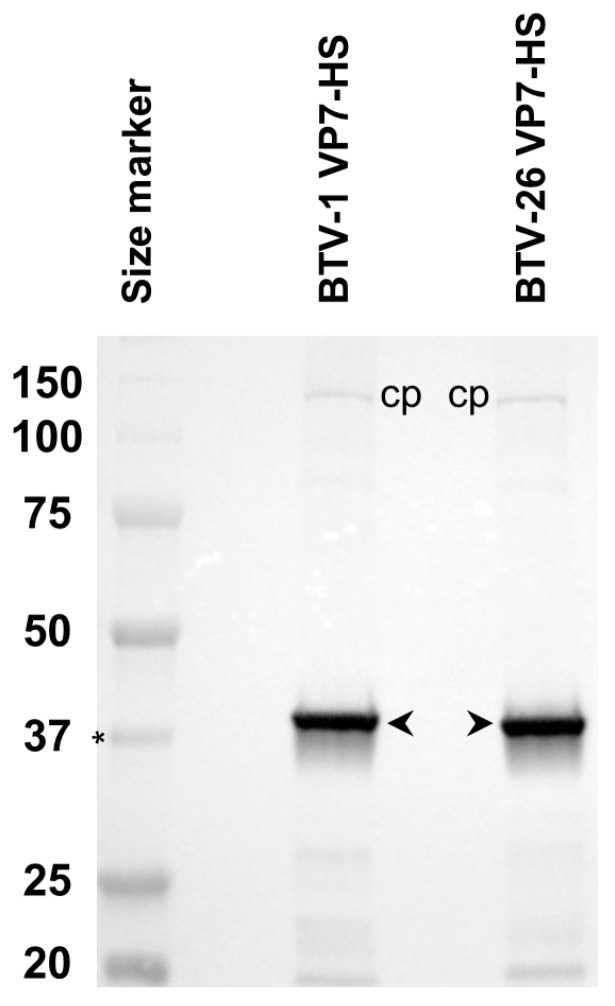
Expression of COOH-terminal tagged VP7 of BTV-1 or BTV-26 in KC cells. KC cells transfected with pKCi-BTV1-VP7-HS and pKCi-BTV26-VP7-HS were harvested at 4 days post-transfection. VP7 of BTV-1 or BTV-26 (indicated by open arrowheads) was expressed as full-length products with sizes compatible with their theoretical molecular weights. The asterisk indicates the position of a 37 kDa size marker band. The intracellular biotinylated protein is indicated as cp.

The expression of COOH-terminal tagged VP7 proteins of BTV-1 and BTV-26 in KC cells transfected with plasmids pKCi-BTV1-VP7-HS and pKCi-BTV26-VP7-HS generated full-length products with comparable expression levels (Figure 13). Similar results were also observed in BSR cells transfected with pCI-BTV1-VP7-HS and pCI-BTV26-VP7-HS or C6/36 cells transfected with pAE-BTV1-VP7-HS and pAE-BTV26-VP7-HS, where VP7 was expressed at high levels.

## 4. Discussion

BTV-26 was isolated from blood samples collected in 2010 in Kuwait, from sheep showing mild clinical signs of bluetongue disease [36]. The virus was shown to replicate in mammalian cells, but not in cells derived from *Culicoides sonorensis* (KC cells) or live *Culicoides* insects [36,37].

BTV-26, similar to other “atypical” BTV serotypes (BTV-25 to BTV-36), appears to be transmitted horizontally from infected to in-contact animals, although the route(s) of infection have not yet been fully characterised. Our previous studies using reassortants containing individual BTV-26 genome segments in a wild-type BTV-1 reference strain ‘backbone’ indicate that Seg-2, -6 and -10 facilitate direct transmission in an IFNAR^(−/−)^ mouse model [22].

In the current study, we used reassortants of BTV-26 and BTV-1RG_C7_ to assess their replication in KC cells. Our results confirm that Seg-1, -2 or -3 of BTV-26 are involved in the replication block of reassortants harbouring these segments in KC cells. Purified virus particles of both BTV-1RG_C7_ and BTV-26 were found to attach to the surface of KC cell incubated on ice, indicating that VP2 proteins of both serotypes can mediate binding to insect cells.

Arthropod cells respond to viral infection by activating innate immune pathways, particularly the RNAi pathway [38], which results in the cleavage and subsequent degradation of viral mRNAs. *Culicoides* KC cells have been shown to respond to BTV infection by producing 21 bp-long viral siRNAs [39], although virus replication is not inhibited. Since BTV-26 fails to replicate in KC cells, we assessed whether Seg-1, -2 or -3 of BTV-26 which appear to be involved in blocking replication in KC cells, are more sensitive to an RNAi response than their BTV-1RG_C7_ homologues. Expression of the mRNAs in cells transfected with *Culicoides* expression plasmids indicates that over a time course up to 52 h post-transfection, the levels of mRNAs steadily increased, with comparable kinetics for RNAs of both BTV-1RG_C7_ and BTV-26. The detection of mRNA synthesis and steady increase in mRNA levels, from homologous constructs of these viruses, suggests that RNA silencing is unlikely to be responsible for the blocking of replication of BTV-26 in KC cells. We have confirmed in this study that BTV-26 replicates at lower levels than BTV-RG_C7_ (1.6 log10 or 96% less) in the Dicer-2 defective C6/36 cells and have previously shown that it replicates also at low levels in Dicer-2 competent U4.4 mosquito cells [16,40].

The VP1s of BTV-1 and BTV-26 expressed in BSR or C6/36 cells have similar sizes, compatible with their full-length theoretical molecular weights. Although both VP1s are synthesised at similar levels in BSR cells, VP1 of BTV-26 was expressed at lower levels in transfected C6/36. Our studies also indicated that the BTV-26 Seg-1 reassortant in the wild-type BTV-1 reference strain backbone replicates in C6/36 cells with an initial lag and produces lower titres as compared to BTV-1 but reaches similar titres by two weeks post-infection. The levels of VP1 expression may therefore be one factor leading to a lower initial efficiency of replication of these reassortants during the infection of C6/36 cells.

The COOH-terminal tagged VP1 proteins of BTV-1 or BTV-26 were both expressed in KC cells, although analysis of expression indicates that VP1 of BTV-26 is expressed at 30% the relative expression level of VP1 of BTV-1. Both NH_2_-terminal tagged VP1 proteins were also expressed in transfected KC cells but were only detected at low levels. The Kozak environment of the NH_2_-terminal tagged VP1s is identical to that used for expressing the NH_2_-terminal tagged VP2 (which is expressed at high levels). This suggests that the lower levels of the NH_2_-terminal tagged VP1 were not simply due to a weak Kozak environment (the sequence surrounding the translation start codon, ATG, is considered to have a strong Kozak environment when it follows the consensus sequence ^A^/_G_NNATGG) suggesting that VP1 may be subject to a rapid turnover in KC cells, potentially due to its capacity to generate dsRNA in presence of cellular dinucleotides [41], which is toxic in eukaryotic cells and in particular in insect cells, leading to activation of the RNAi response [39]. The structural models for VP1 of BTV-1 or BTV-26 both show that the NH_2_ and COOH termini of the VP1 are accessible at the surface, suggesting that the tag is unlikely to affect the fold of the protein.

Although the VP1s of BTV-1 and BTV-26 have nearly identical sizes when expressed in BSR cells, the KC cells-expressed NH_2_-terminal or COOH-terminal tagged VP1 of BTV-26 appears to be ~6–7 kDa larger, as indicated in multiple repetitions of the expression process. It is not clear if this size difference in KC cells arises from specific post-translational modification (PTM) which alters the properties and functionalities of VP1 of BTV-26 in KC cells, as evidenced by the failure of replication of reassortant RGBTV1:1_(26)_ in these cells. Glycosylation of both sites in KC cells would add ~6 kDa to the size of the VP1 of BTV-26, in agreement with the observed size of the expressed protein. A structural model suggests that the addition of a glycan chain made of eight sugars (to Asn 226) could create steric hindrance preventing interaction of the NH_2_-terminal end of a VP3 subunit with the surface of VP1. This particular interaction of the VP3 and the VP1 was previously suggested when the atomic structure of the BTV core was determined by X-ray crystallography [42] and when the structure of the VP1 was determined in situ within the core by cryo-electron microscopy [34]. This hypothesis will be explored in future studies by mutating the potential glycosylation sites in the BTV-26 VP1.

VP2 of BTV-26 was expressed in BSR cells as both a full-length and a 10 kDa shorter form, with comparable levels of synthesis. In C6/36 cells, the shorter form was predominant, and in KC cells only the shorter form was identified. SDS-PAGE and mass spectrometry analyses of BTV-26, purified from infected BSR cells suggested that the VP2 and VP3 comigrate together in two different protein bands [40]. The outer capsid of these BTV-26 particles may therefore contain both long and short forms of the VP2. Transmission electron-microscopy of thin sections from KC cells infected with BTV-26 Seg-2 reassortant in the wild-type BTV1 reference strain backbone identified only BTV core particles [40]. This result suggests that the shorter form of VP2, which is also the sole form expressed in KC cells, failed to form an outer-capsid. Since both VP2 and VP5 proteins are required to simultaneously coat the core particle surface [43] this would prevent outer capsid formation, trapping the progeny virus within the cell cytoplasm, preventing further spread of the infection to other cells.

The smaller VP2 does not appear to be generated by truncation, since tagging is possible at either the -NH_2_ or -COOH termini of the protein. The full-length nature of the BTV-26 VP2 mRNA was confirmed by RT-PCR, excluding splicing of the mRNA as an explanation. The possibility that the shorter form of BTV-26 VP2 is due to a co-translational event that occurs with varying frequencies in different cells and/or hosts was also considered. Possible mechanisms could include ribosome jump/shunt/bypass of highly stable secondary RNA structures. Ribosome jump/shunt/bypass at stem-loop structures has been described in multi-cistronic viral mRNAs to facilitate translation [44,45,46]. The presence of stop codons immediately before or within the sequence of the stem-loop has been described as a key element [44]. Such stop codons cause the ribosomes to pause then jump. This mechanism is well described for several viruses, including plant viruses. However, the ribosome jump in adenovirus occurs in a region that is devoid of any stop codon [45]. This appears to be facilitated by sequence elements in the vicinity of the stem-loop structures [47,48]. Stem-loop structures were identified in the BTV-26 Seg-2 mRNA sequence that have a notably higher GC content and lower MFE values than the homologous regions of BTV-1 and other ‘conventional’ BTV serotypes (BTV-1 to BTV-24). In particular, the first stem-loop structure located between nucleotides 911–1169 has a notably higher GC content (~52%) than the homologous regions in vectored BTVs (43% in case of BTV-1). If a ribosome shunt/jump/bypass occurred at any of these three structures, it would generate a VP2 that is 10–13 kDa shorter than the full-length protein [44,45,46]. Future studies will use redundancy in codon usage to modify the base pairing of these stem-loop structures and hence destabilise the stem-loop structures, while retaining the amino acid sequence of the VP2 protein.

Protein splicing is another mechanism that has been described which can modify protein length, although this mechanism appears to be very specific for organisms with DNA genomes including certain viruses [49,50,51], but it is not known to occur in RNA viruses. It involves an autocatalytic splicing of a protein sequence known as an ‘intein’ at specific sites containing serine, cysteine or threonine amino acids followed by re-ligation of the mature parts of the protein known as ‘exteins’ [49]. Any mutation in these specific sites impairs the process. Seg-2 encodes the most variable protein in each orbivirus species, and BTV VP2 amino acid sequences vary by up to 73% [36]. It is therefore unlikely that protein splicing could explain the existence of the short form of VP2.

In ongoing/future studies, different forms of VP2 of BTV-1 and BTV-26 expressed in BSR and KC cells will be analysed by mass spectrometry to determine their full-length sequences. This will provide evidence for the position at which the potential truncation occurs and insights into the possible mechanism(s) leading to synthesis of a short form of the VP2.

VP3 of BTV-26 and BTV-1 are both expressed well in BSR cells. In C6/36 cells, VP3 of BTV-26 is detected at 3 days post-transfection yet at much lower levels than VP3 of BTV-1. This correlates with the failure of replication of reassortant RGBTV1:3_(26)_ in C6/36 cells with a lag period and then a gradual increase of viral titres. In KC cells, BTV-26 VP3 was not detected at 4 days post-transfection but appeared to accumulate very slowly, becoming detectable only by day 7. This agrees with findings that replication of reassortant RGBTV1:3_(26)_ is blocked in KC cells, with cell culture supernatant virus titres decreasing slightly until day 7 post-infection. Although low levels of BTV-26 VP3 expressed in KC cells from plasmid pKCi-BTV26-SH-VP3 were detected on day 7, virus titres (BTV-26 or reassortant RGBTV1:3_(26)_) continued to decrease in infected KC cells. The VP3 is the main building block which forms the inner layer of the core in which the dsRNA and the transcriptase complex are located [42,52,53]. The results in C6/36 or KC cells suggest that VP3 of BTV-26 is poorly expressed and fails to accumulate, possibly due to specific targeting for degradation and rapid turnover in these cells, reducing the possibility of particle assembly and genome replication.

We did not observe any significant differences in the pattern of expression of VP7 of either BTV-26 or BTV-1, whether in BSR, C6/36 or KC cells. This is compatible with the ability of the RGBTV1:7_(26)_ reassortant to replicate in KC cells although virus titres during the early days post-infection were lower. However, by day 8 post-infection this reassortant reaches similar titres as BTV-1RG_C7_.

## 5. Conclusions

The generation of *Culicoides*-specific expression plasmids will open the way for important studies requiring plasmid-based expression in cells derived from various *Culicoides* species. In summary, all four proteins VP1, VP2, VP3 and VP7 of BTV-1 or BTV-26 are expressed as full-length proteins to levels allowing their detection by Western blot in a mammalian cell line (BSR cells). This agrees with the properties of susceptibility and permissiveness of virus replication in mammalian cell lines for BTV-1 or BTV-26.

In insect cells, combinations of degradation/poor expression and/or protein modification appear to drive the failure of BTV-26 core/whole particles to assemble and replicate effectively. These events were observed in particular in cells derived from the *Culicoides* vector.

## Figures and Tables

**Table 1:** pKCi or pCI-G recombinant expression plasmids containing NH_2_- or COOH-terminal tagged VP1, VP2, VP3 or VP7 ORFs of BTV-1 or BTV-26.

Viral Protein (VP)/Virus	pKCi Derived Plasmids	pCI-G Derived Plasmids
VP1/BTV-26	pKCi-BTV26-SH-VP1 pKCi-BTV26-VP1-HS	pCI-BTV26-VP1-HS
VP2/BTV-26	pKCi-BTV26-SH-VP2 pKCi-BTV26-VP2-HS	pCI-BTV26-SH-VP2
VP3/BTV-26	pKCi-BTV26-SH-VP3	pCI-BTV26-VP3-HS
VP7/BTV-26	pKCi-BTV26-VP7-HS	pCI-BTV26-VP7-HS
VP1/BTV-1	pKCi-BTV1-SH-VP1 pKCi-BTV1-VP1-HS	pCI-BTV1-VP1-HS
VP2/BTV-1	pKCi-BTV1-SH-VP2	pCI-BTV1-SH-VP2
VP3/BTV-1	pKCi-BTV1-SH-VP3	pCI-BTV1-VP3-HS
VP7/BTV-1	pKCi-BTV1-VP7-HS	pCI-BTV1-VP7-HS

**Table 2:** Theoretical molecular weights of VP1, VP2, VP3 and VP7 (native and/or tagged) of BTV-1RG_C7_ or BTV-26.

Virus	Protein Theoretical Molecular Weight in kDa (Native/Tagged)			
	VP1	VP2	VP3	VP7
BTV-1	149.7/153.5	111.9/115.9	103.3/107.1	38.5/42.4
BTV-26	150.0/153.8	110.9/114.9	102.9/106.7	38.5/42.4

**Table 3:** Estimated molecular weights of VP1, VP2, VP3 or VP7 of BTV-1 and BTV-26 estimated from different SDS-PAGE runs for the tagged viral protein expressed in KC cells.

Virus	Protein Estimated Molecular Weight in kDa			
	VP1	VP2	VP3	VP7
BTV-1	141.7	116.2	97.9	38.2
BTV-26	147.9	103.0	97.8	37.4

**Table 4:** GC content and minimum free energy (MFE) calculated for stem-loop structures in regions I, II and III of BTV-26 Seg-2 mRNA.

9.9 kDa Deletion			12.4 kDa Deletion			13.2 kDa Deletion			GC Content of Seg-2
Region I	MFE (kcal/mol)	GC%	Region II	MFE (kcal/mol)	GC%	Region III	MFE (kcal/mol)	GC%	
BTV-1_914-1172	61.8	43.24	BTV-1_1455-1792	81.3	42.07	BTV-1_2290-2635	98.4	44.51	42.65
BTV-4_911-1169	66.6	45.56	BTV-4_1446-1770	79.5	40.92	BTV-4_2275-2620	88.7	44.22	42.84
BTV-6_902-1160	65.8	41.31	BTV-6_1440-1767	78.6	42.07	BTV-6_2272-2617	93.1	42.49	42.43
BTV-9_911-1169	66.8	44.40	BTV-9_1437-1764	78.8	43.60	BTV-9_2272-2617	81.6	38.44	41.70
BTV-26_911-1169	84.7	51.74	BTV-26_1446-1770	90.7	43.08	BTV-26_2278-2623	116.3	45.35	44.02

The GC content and MFE were calculated for stem-loop structures in regions I, II and III of BTV-26 Seg-2 mRNA, as identified by the GC calculator. Although the GC content of the full-length mRNA corresponding to Seg-2 is comparable for all analysed sequences, the GC content is particularly high for region I of BTV-26, as compared to all other stem loops. The MFE is systematically higher for all three stem-loop structures in Seg-2 mRNA of BTV-26, as compared to their homologous regions in BTV-1, -4, -6 or -9. The cells are coloured as a heat map from green (lowest value) to red (highest value).

## Data Availability

All data are presented in the main manuscript and supplementary material.

## Supplementary Materials

The following supporting information can be downloaded at: https://www.mdpi.com/article/10.3390/biom13060878/s1; it includes supplementary text with Figure S1: Schematic representation of PUb gene structure in *Culicoides sonorensis*; Figure S2: EGFP fluorescence in KC cells at 48 h post-transfection; Figure S3: EGFP fluorescence at 48 h post-transfection in C6/36 cells transfected with pAE-EGFP-6xHis or pKCi EGFP-6xHis; Figure S4: Golden gate cloning strategy using SapI restriction enzyme; Figure S5: Design of the pGXz entry plasmid series, which we used to facilitate Goldengate cloning; Figure S6: Real-time PCR Ct values for the mRNAs transcribed from plasmids in KC cells; Figure S7: Comparison of the expression of NH_2_- and COOH-terminal tagged VP1 of BTV-1 or BTV-26 in transfected KC cells; Figure S8: Surface representation and electrostatic potential of the theoretical models for VP1 of BTV1 and BTV-26 generated by Phyre 2; Figure S9: The eight sugar moieties N-Glycan used in modelling the volume occupied by a potential N-glycan linked to Asn 226 of BTV-26 VP1 in Figure 7; Figure S10. Expression of NH_2_-terminal tagged VP3 of BTV-1 or BTV-26 in KC cells; Table S1: Primers used for cloning, sequencing and real-time PCR (promoters, tagged viral ORFs); Table S2: Primers used for the constructions of pGXz entry plasmid series which are described in Figure S5; Table S3: Efficiency, PCR product size and segment position of the real-time PCR primer sets used to quantify Seg-1, -2, -3 and -7 of BTV-1 or BTV-26.;Table S4: Codon usage for Seg-2 of BTV-1, -4, -6, -9 and -26.
