# Supplementary material for: Uncovering the Underlying Mechanisms Blocking Replication of Bluetongue Virus Serotype 26 (BTV-26) in Culicoides Cells
Source: Biomolecules. 2023 May 23;13(6):878. doi: 10.3390/biom13060878 (PMC10296729; doi:10.3390/biom13060878)
Supplement: Supplementary file 1 [file biomolecules-13-00878-s001.zip › biomolecules-2338454-supplementary.pdf]

Supplementary text:

Investigating existing insect expression plasmids in KC cells

A plasmid containing *Drosophila* actin-5C promoter (pACT-Hyg) was kindly provided by Prof. Paul Eggleston (Keel university, UK). EGFP (enhanced green fluorescent protein) was used to replace the hygromycin gene by restriction cloning using enzyme XbaI and SalI, resulting in plasmid pAct5C-EGFP. Another plasmid containing *Aedes aegypti* polyubiquitin promoter, driving expression of EGFP (pKSB(-)-PUb-EGFP) was kindly provided by Dr. Eric Marois (IBMC, Strasbourg). A third plasmid containing the baculovirus promoter OpIE2, driving expression of dsRed (*Discosoma sp.* red fluorescent protein) (pKSB(-)-OpIE2-dsRed) was also provided by Dr. Eric Marois.

These plasmids were used to transfect C6/36 *Aedes albopictus* cells or KC - *Culicoides sonorensis* cells, using Lipofectamine 2000 (Invitrogen, Carlsbad, CA, USA), Metafectene® Pro (Biontex, München, Germany), Insectogene (Biontex, München, Germany) or TransIT®-insect (Mirus Bio, Madison, Wisconsin, USA) as recommended by the manufacturers, in order to assess the strength of the promoter they carry. The intensity of expressed fluorescent proteins was considered as primary reference to assess their efficacy, and were inefficient in KC cells (data not shown).

Construction of expression plasmids

The pCI-neo mammalian expression plasmid was modified in order to be compatible with the Golden Gate cloning method [1] based on the SapI restriction enzyme. The modification consisted in removing the two SapI restriction sites present in the neomycin resistance gene, by cleaving the neomycin ORF using PvuII and BamHI restriction enzymes. This was followed by filling in the BamHI site using Taq polymerase and ligation. The resulting plasmid, designated as pCIΔneo, was then used to insert a Golden Gate cassette. The cassette was obtained by PCR amplifying the Gateway reading frame cassette C.1, containing the chloramphenicol resistance gene and the ccdB gene, without the recombination sites (see Gateway ® Vector Conversion System user guide), using primers MB49 and MB2475 (described in Table S1) containing NheI and NotI restriction sites, respectively. Both primers also contain a SapI restriction site. The PCR products were treated with NheI and NotI then ligated into pCIΔneo double digested with the same enzymes. The resulting plasmid is designated as pCI-G.

The PUb promoter derived from the pKSB(-)-PUb-EGFP plasmid was PCR amplified using primers MB350/MB351 (Table S1). The PCR amplicon was double digested with BglII and NheI to facilitate cloning into pCI-G, generating the plasmid pAE.

Because of the low efficacy of existing insect expression plasmids in KC cells, we constructed a *Culicoides* expression plasmid The PUb promoter is known to drive high levels of expression in homologous cell lines [2]. The amino acid sequence of *Ae. aegypti* PUb was used with the tblastn programme (https://blast.ncbi.nlm.nih.gov/Blast.cgi), to identify the homologous sequence in the *Culicoides* genome/transcriptome. The full-length sequence of PUb was identified in sequence data of Bioproject PRJEB19938 of *Culicoides sonorensis* genome assembly (accession number OGVF03003053.1, scaffold 380). This protein shares 77% amino acid identity with PUb of *Ae. aegypti*. The 5'-NCR of *Culicoides* PUb was used to define the promoter region using Neural Network Promoter Prediction software (https://www.fruitfly.org/seq_tools/promoter.html) [3]. PUb predicted promoter was then PCR amplified (see primers, Table S1) from DNA extracts of KC cells and cloned into plasmid pCI-G after digestion with BglII and NheI restriction enzymes to generate two plasmids containing the PUb promoter (pKC and pKCi). The pKC plasmid contains the sequence of the promoter only, while pKCi contains the sequence of the promoter, the first exon and intron (Figure S1).

The recombinant plasmids were used to transform competent DH5α bacteria. Plasmids were purified from overnight bacterial cultures using the QIAprep plasmid miniprep kit (Qiagen, Les Ulis, France) and were sequenced by Sanger sequencing.

Assessment of expression plasmids functionality on insect cells

Following insertion of EGFP by Golden Gate cloning into pAE, pKC and pKCi, the recombinant plasmids were used to transfect C6/36 or KC cells (as described above), in order to assess the strength of the promoters by fluorescence microscopy (Figure S2 and S3).

The most efficient transfection reagent was TransIT®-insect and the pKCi plasmid generated the highest levels of expression in KC cells, regardless of which transfection reagent was used (Figure S2). *Ae. aegypti* PUb promoter in plasmid pAE is functional in C6/36 cells (Figure S3). Plasmids pKCi-EGFP-6xHis and pAE-EGFP-6xHis were used to transfect C6/36 cells using Lipofectamine 2000 or TransIT®-insect. Cells observed at 48 h post-transfection showed that pAE plasmid generates significantly stronger expression than pKCi (Figure S3) in C6/36 cells. These results indicate that the PUb promoters are most efficient in the cells of the organisms from which they are derived.

Supplementary References:

1. Engler, C.; Kandzia, R.; Marillonnet, S., A one pot, one step, precision cloning method with high throughput capability. PLoS One **2008**, 3, (11), e3647.
2. Anderson, M. A.; Gross, T. L.; Myles, K. M.; Adelman, Z. N., Validation of novel promoter 964 sequences derived from two endogenous ubiquitin genes in transgenic Aedes aegypti. *Insect Mol* 965 *Biol* **2010,** 19, (4), 441-9.
3. Reese, M. G., Application of a time-delay neural network to promoter annotation in the Drosophila melanogaster genome. Comput Chem **2001**, 26, (1), 51-6.

Supplmentary Figures:

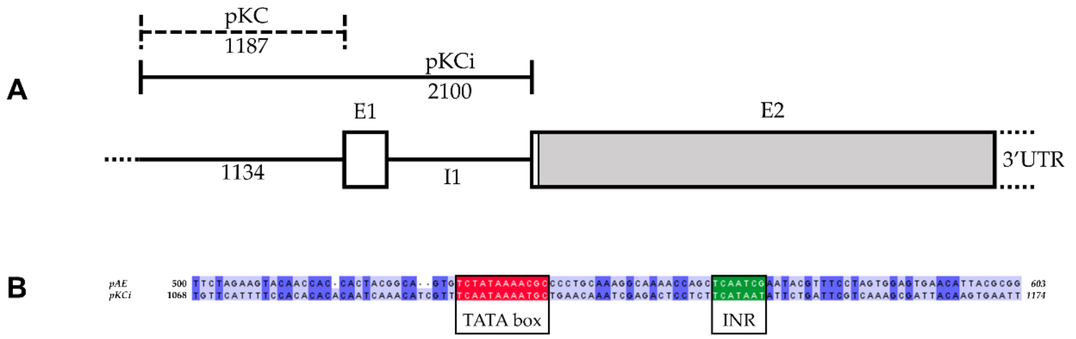

Figure S1. (**A**) Schematic representation of PUb gene structure in *Culicoides sonorensis*. Exons (E) and introns (I) are indicated, with coding sequence (CDS, in grey). Bars above gene represent putative promoter regions used in final expression plasmids. Numbers shown in the promoter region indicate length in base pairs. (**B**) Partial alignment of *Aedes* and *Culicoides* PUb promoters cloned into pAE and pKCi, respectively; visualized and coloured with Jalview. TATA box and initiator element (INR) are boxed.

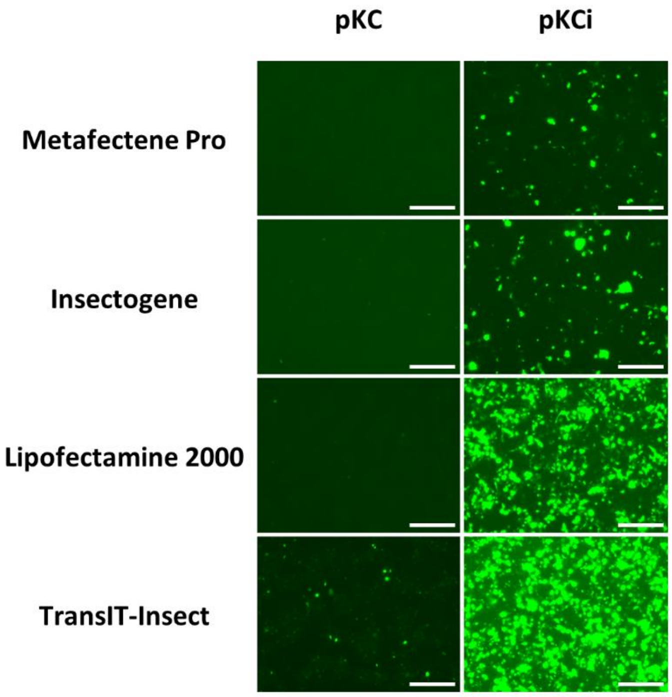

Figure S2. EGFP fluorescence in KC cells at 48 h post-transfection. Expression levels of EGFP under the control of *Culicoides* promoters in plasmids pKC-EGFP-6xHis and pKCi-EGFP-6xHis. KC cells were transfected using Metafectene Pro, Insectogene, Lipofectamine 2000 or TransIT-insect. Scale bar represents 200 μm.

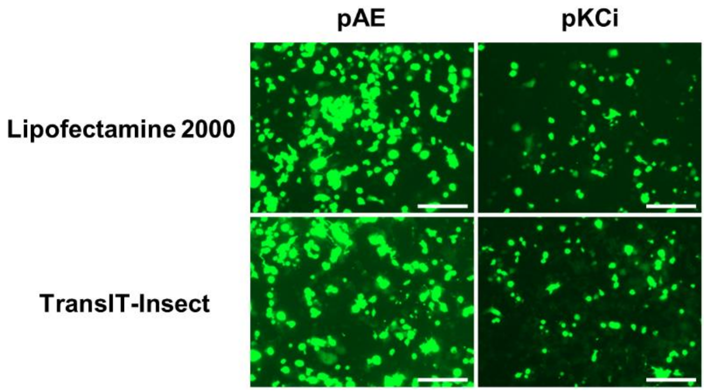

Figure S3. EGFP fluorescence at 48 h post-transfection in C6/36 cells transfected with pAE-EGFP-6xHis or pKCi-EGFP-6xHis. Scale bar represnts 200 μm.

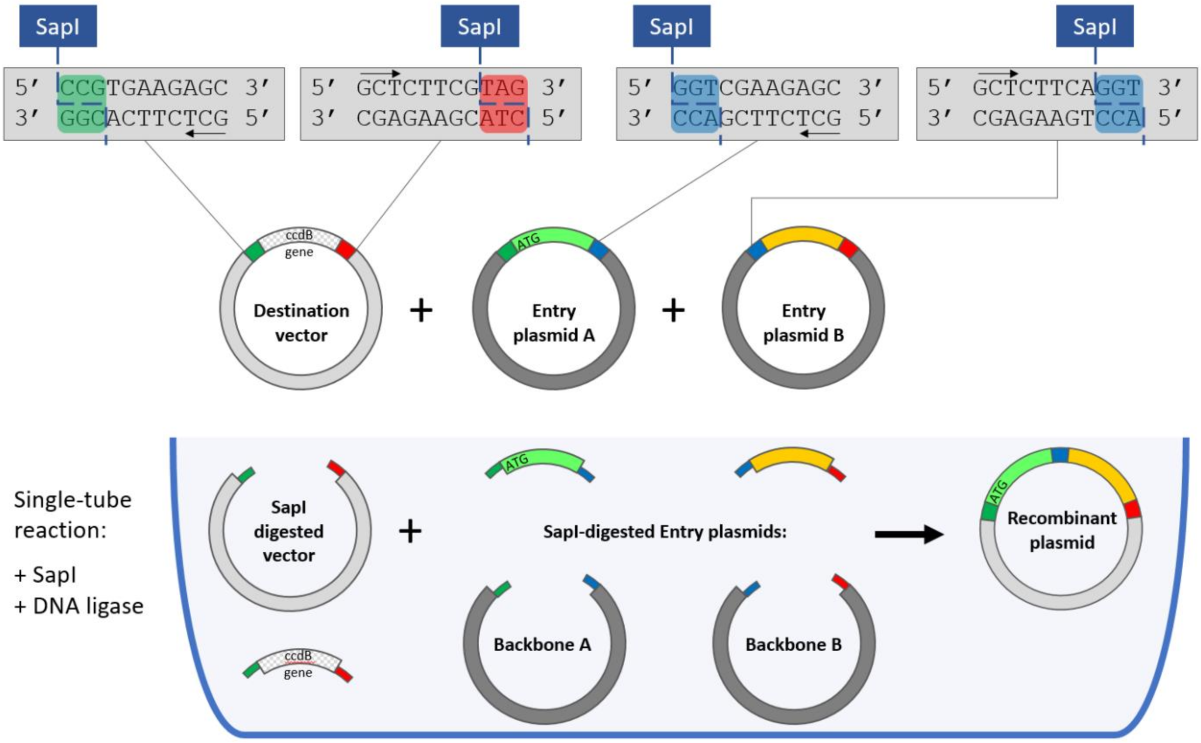

Figure S4. Golden gate cloning strategy using SapI restriction enzyme.

The pGXz entry plasmid series derived from the pDONR/Zeo®

A

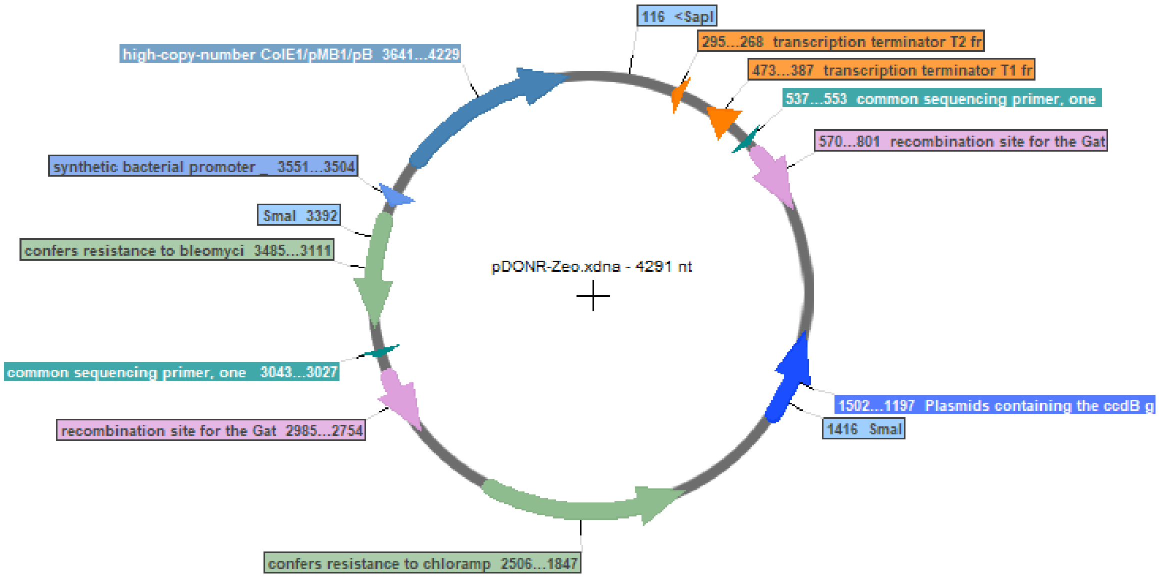

B

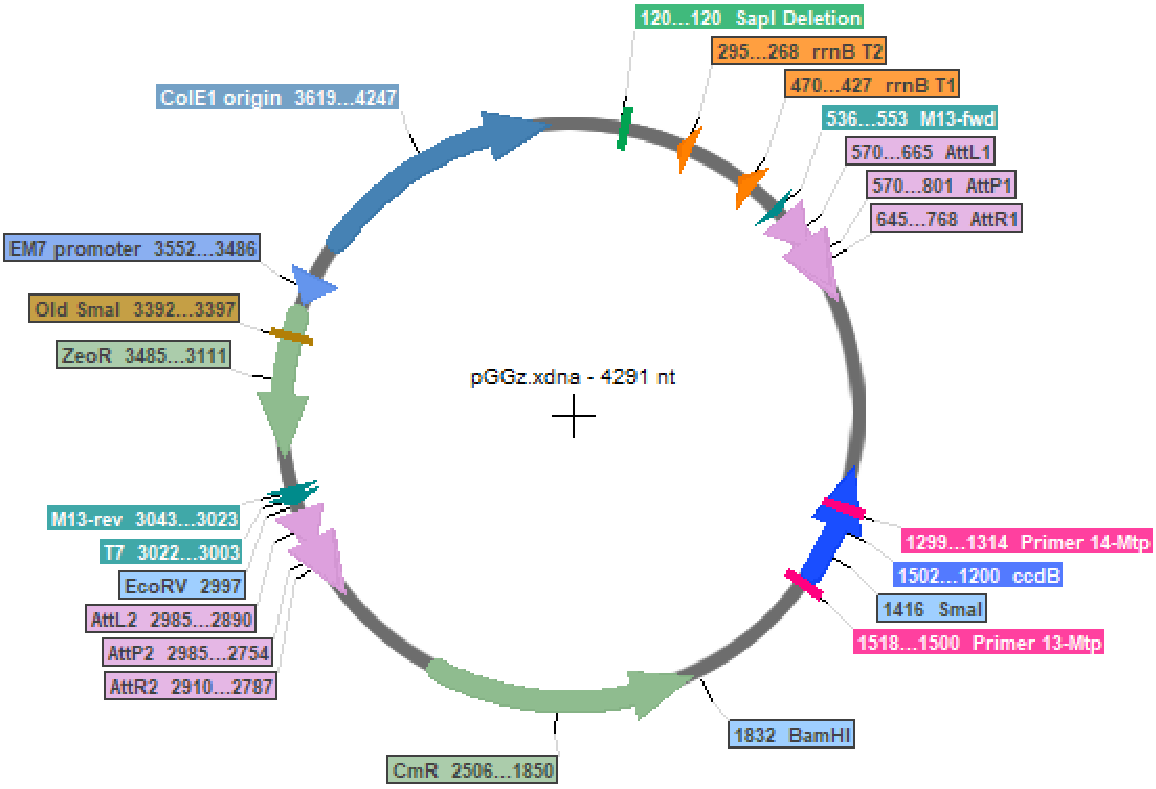

C

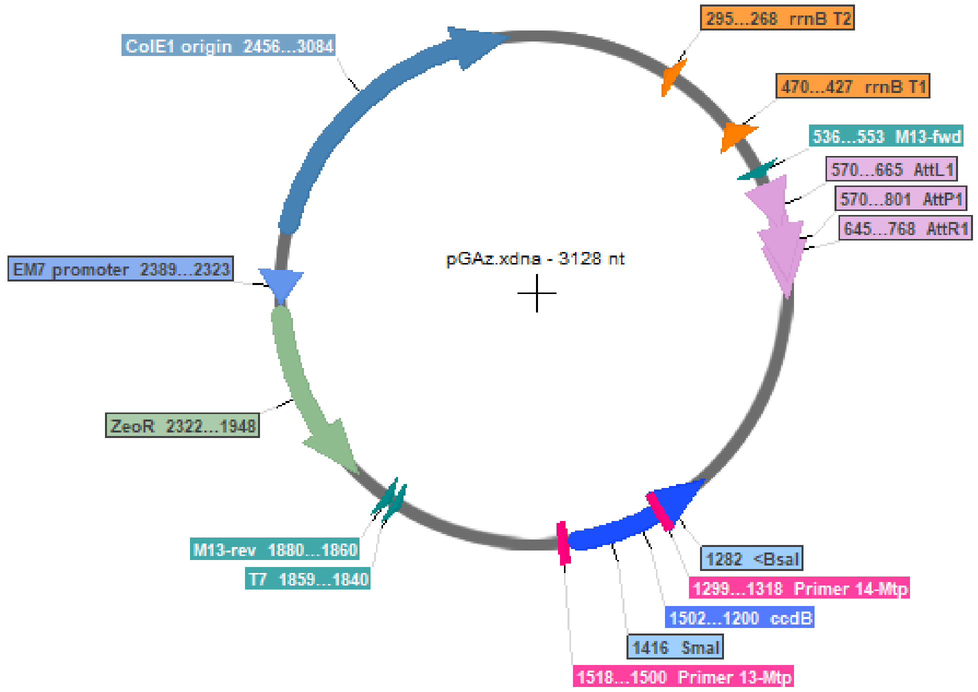
pGAz.xdna - 3128 nt
ColE1 origin 2456...3084
295...268 rrnB T2
470...427 rrnB T1
536...553 M13-fwd
570...665 AttL1
570...801 AttP1
645...768 AttR1
EM7 promoter 2389...2323
ZeoR 2322...1948
M13-rev 1880...1860
T7 1859...1840
1282 <BsaI
1299...1318 Primer 14-Mtp
1502...1200 ccdB
1416 SmaI
1518...1500 Primer 13-Mtp

D

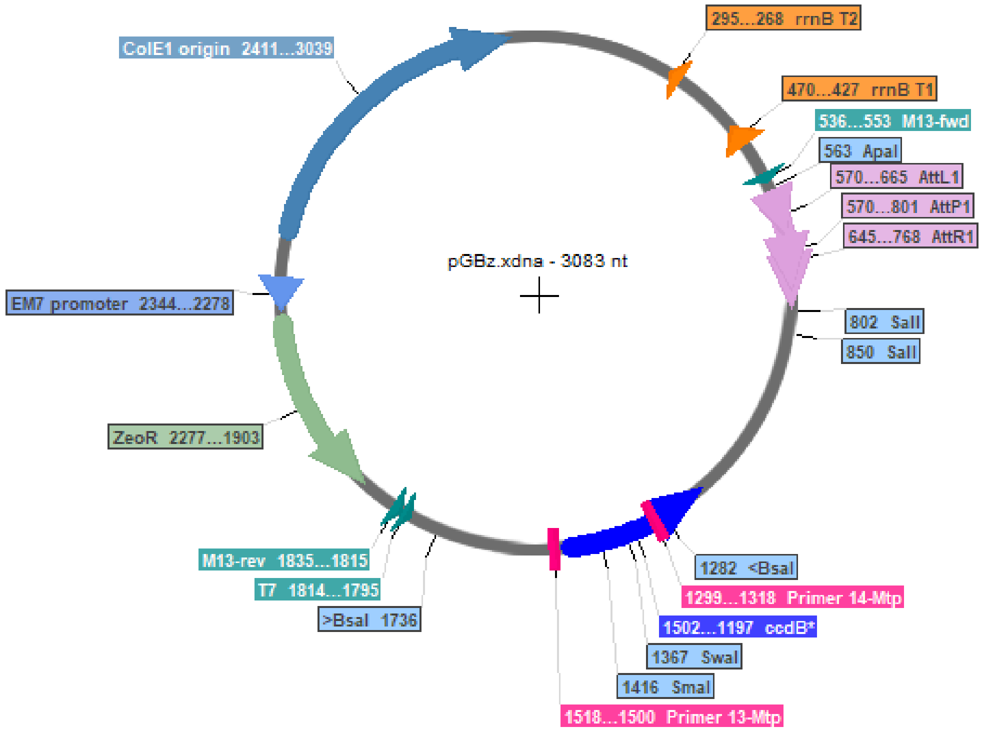
pGBz.xdna - 3083 nt
ColE1 origin 2411...3039
295...268 rrnB T2
470...427 rrnB T1
536...553 M13-fwd
563 ApaI
570...665 AttL1
570...801 AttP1
645...768 AttR1
802 SalI
850 SalI
EM7 promoter 2344...2278
ZeoR 2277...1903
M13-rev 1835...1815
T7 1814...1795
>BsaI 1736
1282 <BsaI
1299...1318 Primer 14-Mtp
1502...1197 ccdB*
1367 SwaI
1416 SmaI
1518...1500 Primer 13-Mtp

E

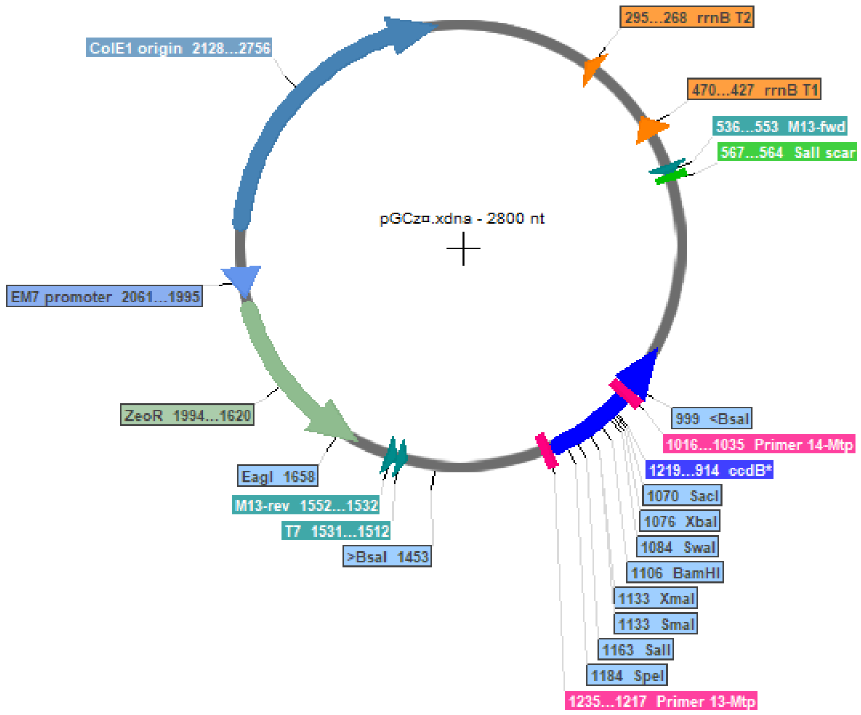
pGCz¤.xdna - 2800 nt
295…268 rrnB T2
470…427 rrnB T1
536…553 M13-fwd
567…564 SalI scar
ColE1 origin 2128…2756
EM7 promoter 2061…1995
ZeoR 1994…1620
EagI 1658
M13-rev 1552…1532
T7 1531…1512
>BsaI 1453
999 <BsaI
1016…1035 Primer 14-Mtp
1219…914 ccdB*
1070 SacI
1076 XbaI
1084 SwaI
1106 BamHI
1133 XmaI
1133 SmaI
1163 SalI
1184 SpeI
1235…1217 Primer 13-Mtp

F

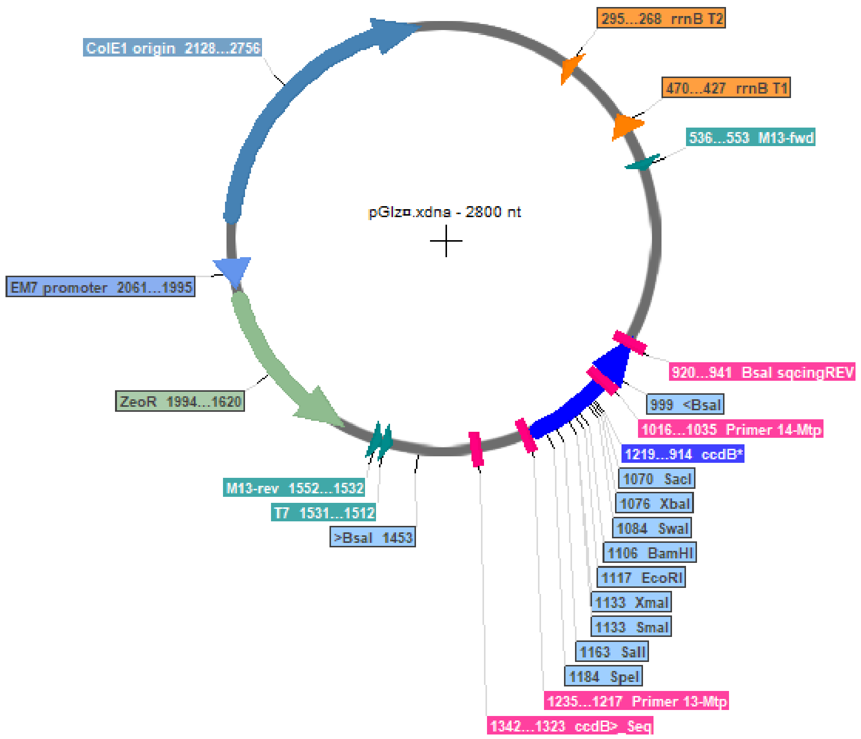
pGIz¤.xdna - 2800 nt
295…268 rrnB T2
470…427 rrnB T1
536…553 M13-fwd
ColE1 origin 2128…2756
EM7 promoter 2061…1995
ZeoR 1994…1620
M13-rev 1552…1532
T7 1531…1512
>BsaI 1453
920…941 BsaI sqcingREV
999 <BsaI
1016…1035 Primer 14-Mtp
1219…914 ccdB*
1070 SacI
1076 XbaI
1084 SwaI
1106 BamHI
1117 EcoRI
1133 XmaI
1133 SmaI
1163 SalI
1184 SpeI
1235…1217 Primer 13-Mtp
1342…1323 ccdB>_Seq

G

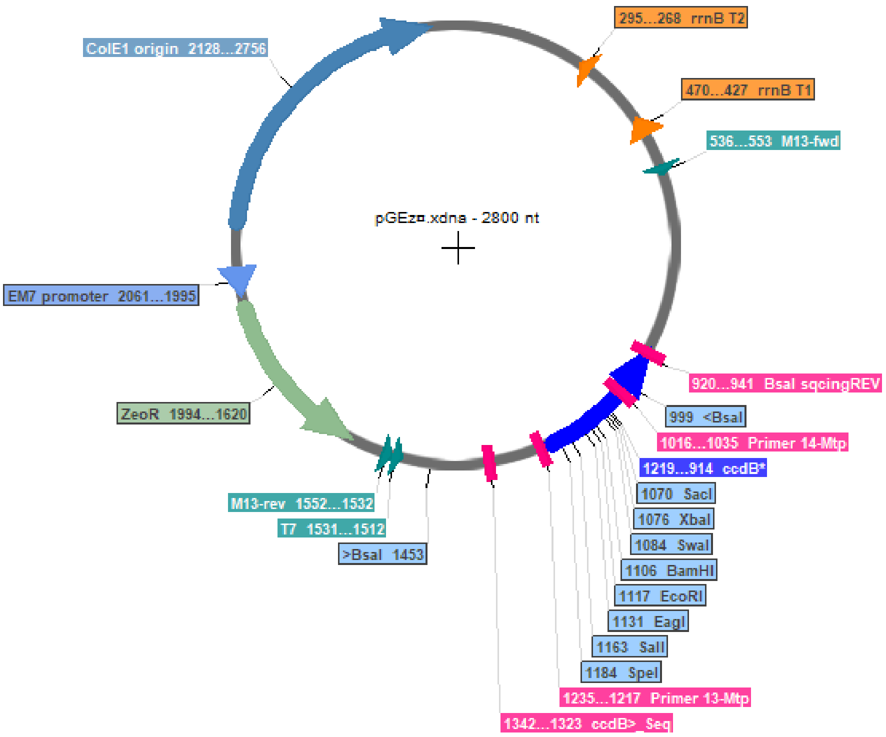

Figure S5. Design of the pGXz entry plasmid series, which we used to facilitate Goldengate cloning. A brief description of how the plasmids were generated from the pDONR/Zeo is provided. Primers used for the constructions are described in Table S3. (**A**) original map of entry plasmid pDONR/Zeo. (**B**) pGGz entry plasmid was constructed in 2015 at IBMP (Institut de biologie moléculaire des plantes, CNRS, Strasbourg, France). This plasmid was derived from pDONR/Zeo by PCR amplifying the latter using primers which permitted mutation of SapI site (downstream ColE1 origin of replication, position 116) and mutation of SmaI (within the zeocine resistance gene, at position 3392). Plasmid pGGz contains another SmaI site in the ccdB ORF. This SmaI site is used for blunt-end cloning of PCR products containing SapI sites which are included in PCR primers. Disruption of the ccdB ORF allows standard E. coli strains to grow on LB-agar plate containing zeocine and/or chloramphenicol. (**C**) pGAz plasmid results from downsizing of pGGz plasmid by deleting EcoRV-BamHI fragment (containing CmR gene and AttL2, AttP2, AttR2 recombination sites). pGAz was constructed in 2016 at UMR BGPI (Biologie et Génétique des interactions Plantes-parasites pour la Protection Intégrée, Montpellier, France). (**D**) pGBz is derived from plasmid pGAz by addition of an SwaI restriction site into the ccdB ORF and an additional BsaI site (position 1736). Therefore, the two BsaI restriction sites in pGBz allow cloning of PCR amplicon containing BsaI sites which have been previously included in the PCR primers. (**E**) pGCz¤ plasmid resulted from downsizing of pGBz by removing the ApaI-SalI fragment (containing AttL1, AttP1 and AttR1 recombination sites). This was followed by introducing an MCS (containing sites SpeI, SalI, BamHI, XbaI and SacI) into ccdB by PCR. Therefore, the final MCS within the ccdB of pGCz¤, contains sites SpeI, SalI, SmaI, BamHI, SwaI, XbaI, SacI and BsaI. (**F**) pGIz¤ plasmid resulted from the insertion of an EcoRI restriction site by PCR into MCS between SmaI and BamHI. (**G**) pGEz¤ plasmid resulted from a mutagenesis by PCR of pGCz¤, by first deleting the EagI restriction site within the zeocine resistance gene. This was followed by replacing SmaI site of pGCz¤ by EagI which facilitates cloning of DNA fragments containing EagI and/or NotI.

¤ in pGCz, pGIz and pGEz denotes a TAG (Amber codon) in the ccdB immediately following the ATG start. The presence of this Amber codon necessitates the use an Amber suppressor *E. coli* strain, such as DH5-*α*.

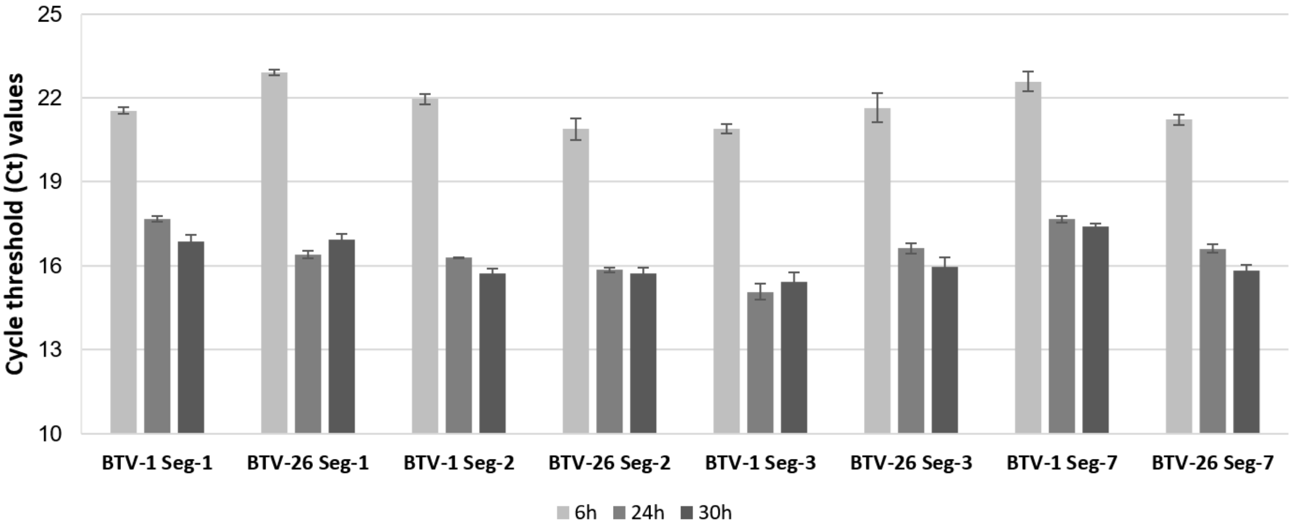

Figure S6. Real-time PCR Ct values for the mRNAs transcribed from plasmids in KC cells.Plasmids pKCi-BTV1-VP1-HS, pKCi-BTV26-VP1-HS, pKCi-BTV1-SH-VP2, pKCi-BTV26-SH-VP2, pKCi-BTV1-SH-VP3, pKCi-BTV26-SH-VP3, pKCi-BTV1-VP7-HS or pKCi-BTV26-VP7-HS were transfected into KC cells. The RNA extracts were reverse transcribed using hexanucleotide primers followed by PCR using primers described in Table S1. The results indicate that levels of mRNAs increased over the time course between 6 hours and 30 hours post-transfection.

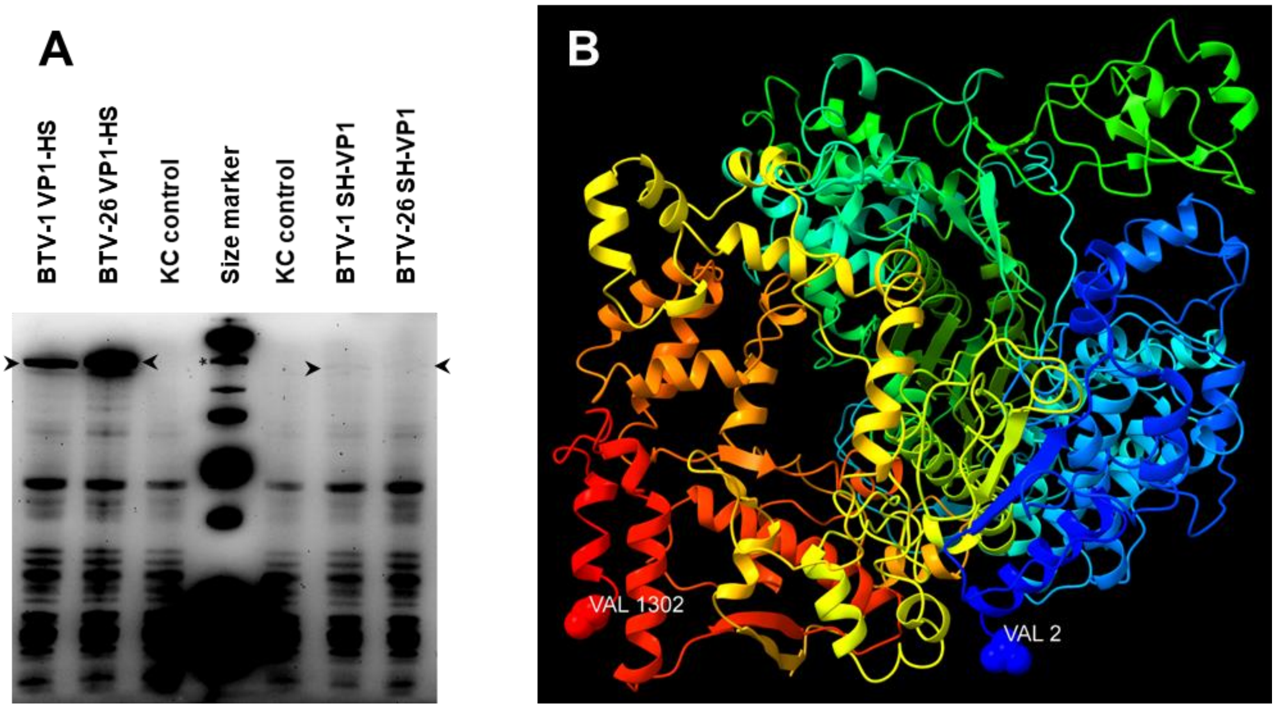

Figure S7. Comparison of the expression of NH_2- and COOH-terminal tagged VP1 of BTV-1 or BTV-26 in transfected KC cells. (**A**) the VP1 proteins of both BTV-1 and BTV-26 are expressed as full-length products (indicated by open arrowheads). The asterisk indicates the position of the 150 kDa size marker band. The NH_2-terminal tagged proteins are detected at low levels. (**B**) a rainbow coloured cartoon representation of the models of VP1 of BTV-1 and BTV-26 showing the NH_2 and COOH termini accessible at the surface.

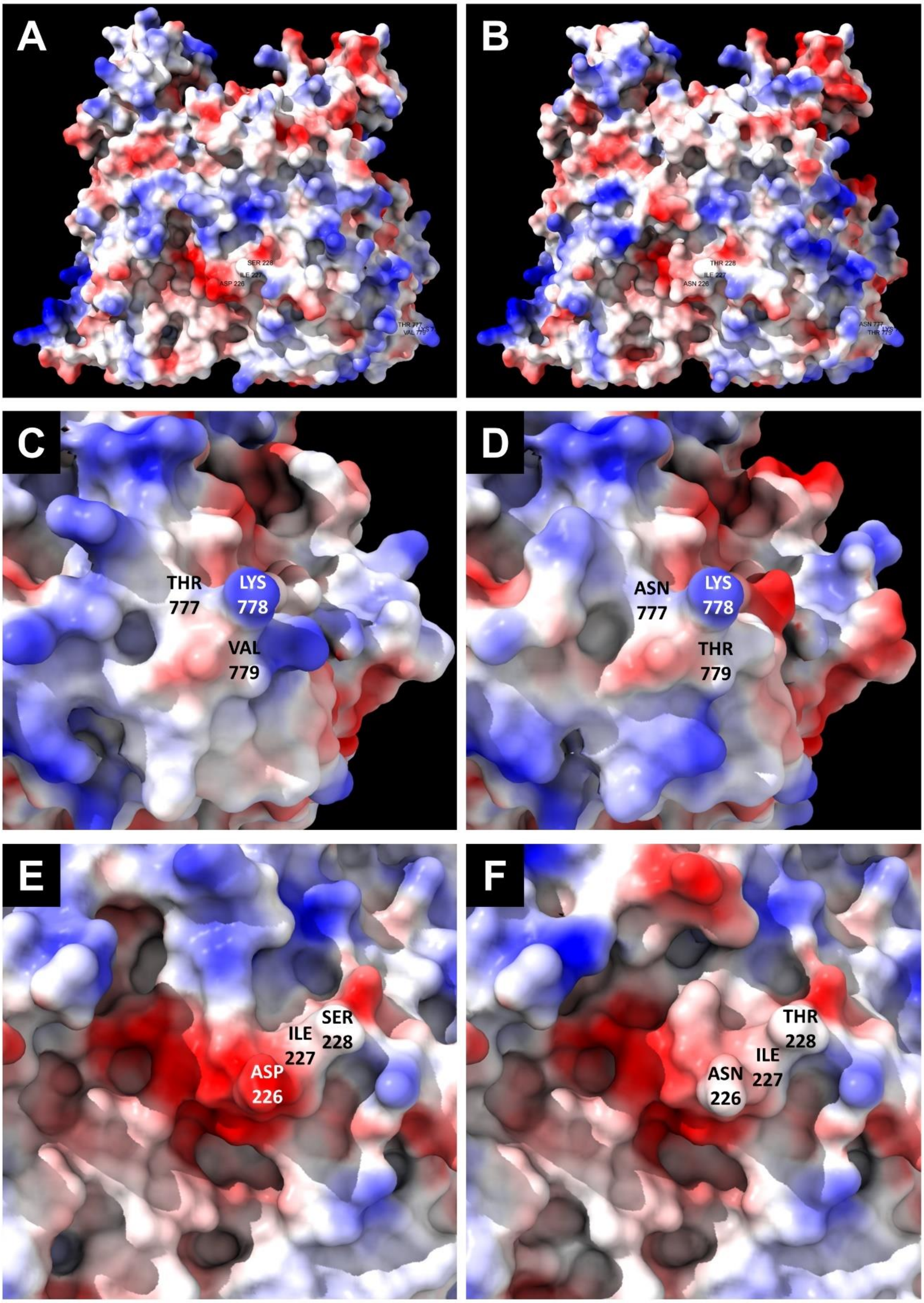

Figure S8. Surface representation and electrostatic potential of the theoretical models for VP1 of BTV-1 and BTV-26 generated by Phyre 2. **(A**) and **(B**) structural models of the whole VP1 of BTV-1 and BTV-26, respectively. (**C**) and **(D**) the region containing sites at position 777-779 of the VP1 amino acid sequence for BTV-1 (TKV) and BTV-26 (NKT), respectively. (**E**) and **(F**) the region containing the sites at position 226-228 of the VP1 amino acid sequence for BTV-1 (DIS) and BTV-26 (NIT), respectively. Asn 226 and 777 in BTV-26 VP1 are predicted as potentially glycosylated.

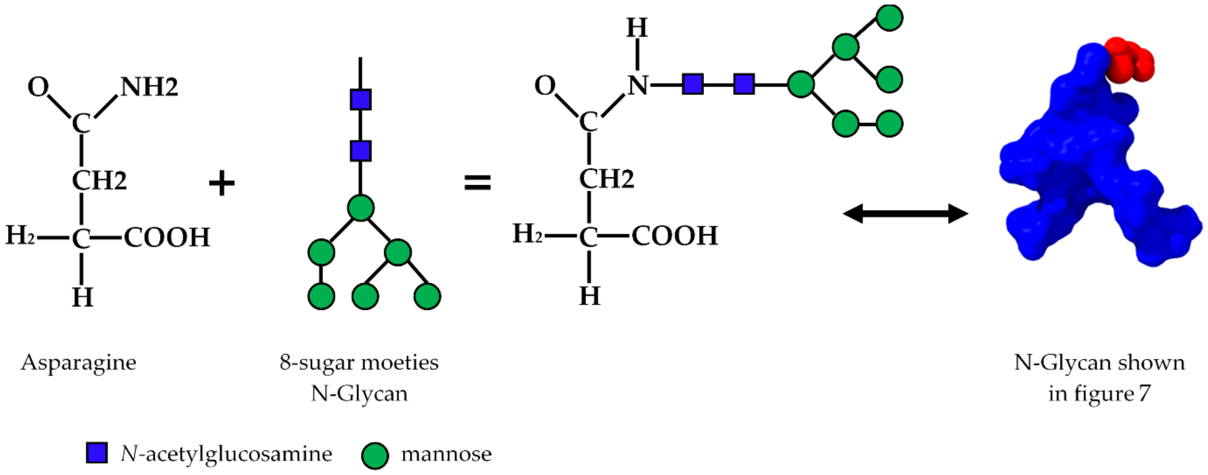

Figure S9. The 8 sugar moieties N-Glycan used in modelling the volume occupied by a potential N-glycan linked to Asn 226 of BTV-26 VP1 in Figure 7. The nomenclature of this N-glycan is described as: alpha-D-manopyranose-(1-2)-alpha-D-manopyranose-(1-3)-[alpha-D-manopyranose-(1-3)-[alpha-D-manopyranose-(1-6)]alpha-D-manopyranose-(1-6)]alpha-D-manopyranose-(1-4)-2-acetamido-2-deoxy-beta-D-glucopyranose-(1-4)-2-acetamido-2-deoxy-beta-D-glucopyranose.

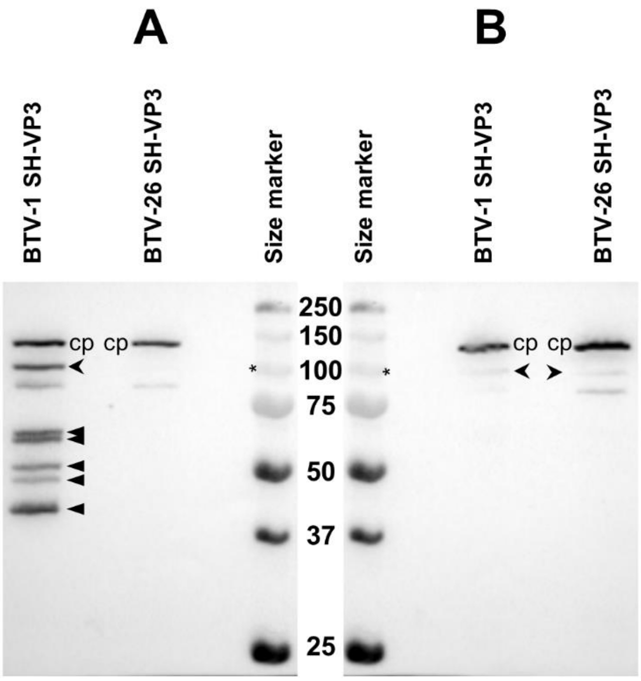

Figure S10. Expression of NH_2-terminal tagged VP3 of BTV-1 or BTV-26 in KC cells. Expression and quantification of NH_2-terminal tagged VP3 of BTV-1 or BTV-26 in KC cells harvested at 4 or 7 days post-transfection with pKCi-BTV26-SH-VP3 or pKCi-BTV1-SH-VP3. Western blots were performed in absence of biotin blocking buffer. The intracellular biotinylated protein (indicated as cp) was used as loading control. (**A**) At 4 days post-transfection, VP3 of BTV-1 (indicated by open arrowhead) is expressed as full-length product with a size compatible with its theoretical molecular weight. Degradation products of BTV-1 VP3 were observed at day 4 post-transfection and are indicated by closed arrowheads. VP3 of BTV-26 was not detected in cells harvested at 4 days post-transfection. (**B**) In cells harvested at 7 days, VP3 of BTV-1 and BTV-26 (indicated by open arrowheads) were both detected (ratio 5:3). Asterisk indicates the position of the 100 kDa size marker band.

Table S1. Primers used for cloning, sequencing and real-time PCR (promoters, tagged viral ORFs).

Primer	**Sequence (5'- to -3')** including 5' extension, **SapI** restriction sites	Target	Aim
MB49	TGCTGCTAGCCCGTGAAGAGCTTAGGCACCCCAGGCTT	Gateway cassette C.1	pCI-G plasmid
MB247	AGATGCGGCCGCCTACGAAGAGCACTGGCTGTGTATAAGGGA	Gateway cassette C.1	pCI-G plasmid
MB353	AGTCAGATCTTGGGAAAGTTCAAGAGACGGAATATTC	PUb *Culicoides*	Cloning promoter: pKC & pKCi
MB311	ATCTGCTAGCGTTTTCTCTTTAAAATTCACTTGTAATCG	PUb *Culicoides*	Cloning promoter (w/o intron): pKC
MB312	ATCTGCTAGCTATGTCTGTAATGAGATTAAAAAAATACGCTAT	PUb *Culicoides*	Cloning promoter (with intron): pKCi
MB350	AGTCAGATCTATCTTTACATGTAGCTTGTGCATTGAATC	PUb *Aedes*	Cloning promoter: pAE
MB351	ATCTGCTAGCGTTGAAATCTCTGTTGAGCAGAAAAAG	PUb *Aedes*	Cloning promoter: pAE
MB193	CAGGGTCGGAACAGGAGAG	pCIneo (backbone)	Sequencing (novel promoters)
MB92	CATTCTAGTTGTGGTTTGTC	pCIneo (backbone)	Sequencing (novel promoters)
MB381	TTCCACACACACAATCAAACATCG	pKC	Sequencing
MB382	GGCAGATTATTCTGAAGTTAGATAAGG	pKCi	Sequencing
MB448	TGAGAGTTTGCATTTCACAACC	pAE	Sequencing
MB160	ATTA**GCTCTTC**ACC*GTCAAC*ATGCATCACCATCACCATCACGGTTGAAGAGCATTA	6xHis-tag (H)	GoldenGate entry clone (with *Kozak*+ATG)
MB161	TAAT**GCTCTTC**AACCGTGATGGTGATGGTGATGCAT*GTTGAC*GGTGAAGAGCTAAT	6xHis-tag (H)	GoldenGate entry clone (with *Kozak*+ATG)
MB425	ATTA**GCTCTTC**AGGTCATCACCATCACCATCACAGT	6xHis+TwinStrep-Tag (HS)	GoldenGate entry clone (with Stop)
MB426	AAT**GCTCTTC**ACTATTACTTCTCAAATTGTGGATGAGACCA	6xHis+TwinStrep-Tag (HS)	GoldenGate entry clone (with Stop) RT
MB427	ATAGGATCC**GCTCTTC**GCC*GTCAAC*ATGAGTGCTTGGAGTCAT	TwinStrep+6xHis-Tag (SH)	GoldenGate entry clone (with *Kozak*+ATG)
MB428	TATGGATCC**GCTCTTC**GACCGTGATGGTGATGGTGATGCTTCT	TwinStrep+6xHis-Tag (SH)	GoldenGate entry clone (with Kozak+ATG)
MB254	ATTA**GCTCTTC**AGGTCATCACCATCACCATCACTAATAGTGAAGAGCATT	6xHis-tag (H)	GoldenGate entry clone (with Stop)
MB255	AAT**GCTCTTC**ACTATTAGTGATGGTGATGGTGATGACCTGAAGAGCTAAT	6xHis-tag (H)	GoldenGate entry clone (with Stop)
MB306	ATAGCCG**GCTCTTC**ACCGGTCAACATGGTGAGCAAGGGCGAGGA	EGFP	GoldenGate entry clone (w/o Stop)
MB307	ATAGCCG**GCTCTTC**GACCGGATCCCTTGTACAGCTCGTCCA	EGFP	GoldenGate entry clone (w/o Stop)
MB55	ATAG**GCTCTTC**ACCGAATGCAATGGTCGCAATCACCGTGC	BTV-1 VP1	GoldenGate entry clone (w/o Stop; for C-ter fusion)
MB56	ATAT**GCTCTTC**AACCAACGAATTGGTTTTCAAGATTTCTAAC	BTV-1 VP1	GoldenGate entry clone (w/o Stop; for C-ter fusion)
MB328	ATCTAGCGGCC**GCTCTTC**AGGTATGGTCGCAATCACCGTGCA	BTV-1 VP1	GoldenGate entry clone (for N-ter fusion)
MB329	ACTATGCGGCC**GCTCTTC**ACTAAACGAATTGGTTTTCAAGATTTCTAAC	BTV-1 VP1	GoldenGate entry clone (for N-ter fusion)

Primer	**Sequence (5'- to -3')** including 5' extension, **SapI** restriction sites	**Target**	**Aim**
MB102	ATGGTCTC**GCTCTTC**ACCGAATGCAATGGTCGCGATTACCG	BTV-26 VP1	GoldenGate entry clone (w/o Stop; for C-ter fusion)
MB103	ATGGTCTCATAAC**GCTCTTC**AACCAACGAATTGGTTCTCTAAGCCCCTTAC	BTV-26 VP1	GoldenGate entry clone (w/o Stop; for C-ter fusion)
MB178	ATGGTCTC**GCTCTTC**AGGTATGGTCGCGATTACCG	BTV-26 VP1	GoldenGate entry clone (for N-ter fusion)
MB251	ATGGTCTCATAAC**GCTCTTC**ACTATCATCAAACGAATTGGTTCTCTAAGCCCCTT	BTV-26 VP1	GoldenGate entry clone (for N-ter fusion)
MB334	ATAGGATCC**GCTCTTC**GGGTATGGATGAACTAGGCATCCCAGTTTA	BTV-1 VP2	GoldenGate entry clone (for N-ter fusion)
MB335	TATGGATCC**GCTCTTC**GCTATCATACGTTGAGAAGTTTTGTTAGCATCT	BTV-1 VP2	GoldenGate entry clone (for N-ter fusion)
MB324	ATAGGATCC**GCTCTTC**GCCGTCCACCATGGAGGAATTTGTCATTCCCAT	BTV-26 VP2	GoldenGate entry clone (w/o Stop; for C-ter fusion) RT-PCR
MB325	TATGGATCC**GCTCTTC**GACCCACGTTGAGCAACTTTGTCATC	BTV-26 VP2	GoldenGate entry clone (w/o Stop; for C-ter fusion) RT-PCR
MB340	ATAGGATCC**GCTCTTC**GGGTATGGAGGAATTTGTCATTCCCATAT	BTV-26 VP2	GoldenGate entry clone (for N-ter fusion)
MB341	TATGGATCC**GCTCTTC**GCTACTACACGTTGAGCAACTTTGTCATCAATT	BTV-26 VP2	GoldenGate entry clone (for N-ter fusion)
MB336	ATCTAGCGGCC**GCTCTTC**AGGTATGGCTGCTCAGAATGAGCAACGT	BTV-1 VP3	GoldenGate entry clone (for N-ter fusion)
MB337	ACTATGCGGCC**GCTCTTC**ACTACACAGTTGGCGCAGCCA	BTV-1 VP3	GoldenGate entry clone (for N-ter fusion)
MB342	ATAGGATCC**GCTCTTC**GGGTATGGCTGCTCAAGATGAGCGCAA	BTV-26 VP3	GoldenGate entry clone (for N-ter fusion)
MB343	TATGGATCC**GCTCTTC**GCTACTACGCAGTCGGCGCAGCTAG	BTV-26 VP3	GoldenGate entry clone (for N-ter fusion)
MB242	ATGGTCTC**GCTCTTC**ACCGATAGAGATGGACACTATCGCTGCAAG	BTV-1 VP7	GoldenGate entry clone (w/o Stop; for C-ter fusion)
MB243	ATGGTCTCATAAC**GCTCTTC**AACCCACATAGGCGGCGCGTG	BTV-1 VP7	GoldenGate entry clone (w/o Stop; for C-ter fusion)
MB332	ATAGGATCC**GCTCTTC**GCCGATAGAGATGGACACTATCGCAGCGA	BTV-26 VP7	GoldenGate entry clone (w/o Stop; for C-ter fusion)
MB333	TATGGATCC**GCTCTTC**GACCTACATAAGCGGCACGAGCAATC	BTV-26 VP7	GoldenGate entry clone (w/o Stop; for C-ter fusion)
MB286	GACCAGAACTTATCTCCGCAG	BTV-1 Seg-1	Real-time PCR
MB432	GCATAACGACATCCTTTCTC	BTV-1 Seg-1	Real-time PCR
MB288	AACCCATGTGATTTATACCCAG	BTV-1 Seg-2	Real-time PCR
MB289	ACTGTTCTTGATTCCAACCC	BTV-1 Seg-2	Real-time PCR
MB294	AGAGTCCAAGTCAATTATGGTC	BTV-1 Seg-3	Real-time PCR
MB295	TGTAGCCCATCCATTATATCCT	BTV-1 Seg-3	Real-time PCR
MB296	GGGTAACTCACAGCAAACTC	BTV-1 Seg-7	Real-time PCR
MB297	AAGGCAGGGTATTGATTTAAGG	BTV-1 Seg-7	Real-time PCR

**Primer**	**Sequence (5'- to -3')** including 5' extension, **SapI** restriction sites	**Target**	**Aim**
MB437	TGTTTCCAGACCAGAATTTATCCC	BTV-26 Seg-1	Real-time PCR
MB438	TGTTTGCCGTTATGAATCCAC	BTV-26 Seg-1	Real-time PCR
MB273	TCTATTATGATTTGCACCCGCT	BTV-26 Seg-2	Real-time PCR
MB274	GCCGTCAATGTATAGTTCGTC	BTV-26 Seg-2	Real-time PCR
MB441	GAACCTACACATAGAAACGACTC	BTV-26 Seg-3	Real-time PCR
MB442	CTATCATTTAGAACAGCCTTCCAG	BTV-26 Seg-3	Real-time PCR
MB281	CAGTTAGTGTCGGTGGTGTG	BTV-26 Seg-7	Real-time PCR
MB282	ATAGAACGCAACTTGAATCTGG	BTV-26 Seg-7	Real-time PCR

Table S2. Primers used for the constructions of pGXz entry plasmid series which are described in Figure S5.

Primer	Sequence (5'- -3')	Aim	Matrix	Product
BM_MutSmaI-pDz<	GGTTCTCaCGaGACTTCGTGGAGGA	Mutation of SmaI restriction site within the zeocine resistance gene	pDONR/Zeo	pDONR/Zeo ΔSmaIZeo
BM_pDz-3404>	CGAGCCGGTCGGTCCAGA	Mutation of SmaI restriction site within the zeocine resistance gene	pDONR/Zeo	pDONR/Zeo ΔSmaIZeo
BM_MutSapI-ColE1<	AAGCGGAAGtGCGCCCAATAC	Mutation of SapI restriction site downstream ColE1 origin of replication	pDONR/Zeo ΔSmaIZeo	pGGz
BM_ColE1>	CCTCGCTCACTGACTCGCTG	Mutation of SapI restriction site downstream ColE1 origin of replication	pDONR/Zeo ΔSmaIZeo	pGGz
pGAz-BsaI<	CaagagCGCGCAAATACGCATACTGTTATC	Addition of a 2nd BsaI restriction site upstream ccdB; mutagenesis	pGAz	pGAzb
pGAz-BsaI>	AGACCAGAGGTATGCTATGAAGCAGCGTATT	Addition of a 2nd BsaI restriction site upstream ccdB; mutagenesis	pGAz	pGAzb
ccdB-D44F>	ttTAAAtTgTCCCGTGAACTTTACCC	Addition of an SwaI restriction site into ccdB ORF; mutagenesis	pGAzb	pGBz
ccdB-AA43-38_Rev	TGACAGCAGACGTGCACTG	Addition of an SwaI restriction site into ccdB ORF; mutagenesis	pGAzb	pGBz
Ins_SpeISalI-ccdB_F	AGAactAGtCGTTATCGTCTGTTTGTcGAcGTACAGAGTGATATTATTG	Amplification, insertion of an MCS (SpeI, SalI, XbaI, SacI) into ccdB ORF	pGBz	pGSz¤
Ins_SacIXbaI-ccdB_R	GTAgAGcTCtCtaGAcAATTTAAATGACAGCAGACGTGCACT	Amplification, insertion of an MCS (SpeI, SalI, XbaI, SacI) into ccdB ORF	pGBz	pGSz¤
Vec_SacI-ccdB_F	taGaGAgCTcTACCCGGTGGTGCATATC	Amplification of pGBz (ΔApaI-SalI fragment), ligation with MCS insert	pGBz ΔApaI-SalI fragment	pGSz¤
Vec_SpeI-ccdB_R	AACGaCTagtTCTTTTATAGGTGTAAACCTTAAACTGCAT	Amplification of pGBz (ΔApaI-SalI fragment), ligation with MCS insert	pGBz ΔApaI-SalI fragment	pGSz¤
Ins_BamHI-ccdB_F	CACGTCTGCTGTCAttTAAAtTgTC	Addition of an BamHI restriction site into ccdB ORF; mutagenesis	pGSz¤	pGCz¤
Ins_BamHI-ccdB_R	CggatccCAGGGGGATCACCATCCGT	Addition of an BamHI restriction site into ccdB ORF; mutagenesis	pGSz¤	pGCz¤
Zeo -EagI_F	CAACTGCGTGCACTTCGTG	Mutation of EagI restriction site within the zeocine resistance gene	pGCz¤	pGCz¤ ΔEagI
Zeo -EagI_R	CCtGCtGGGTCGCGCAG	Mutation of EagI restriction site within the zeocine resistance gene	pGCz¤	pGCz¤ ΔEagI
Ins_EagI-ccdB_F	cCGACGGATGGTGATCCC	Addition of an EagI restriction site into ccdB ORF; mutagenesis	pGCz¤ ΔEagI	pGεz¤
Ins_EagI-ccdB_R	CCGGGCGTGTCAATAATATCAC	Addition of an EagI restriction site into ccdB ORF; mutagenesis	pGCz¤ ΔEagI	pGεz¤
Ins_EcoRI-ccdB_F	attagctcttcggaATtCCCCTGggatccGCACGTCTG	Addition of an EcoRI restriction site into ccdB ORF; mutagenesis	pGCz¤, pGεz¤	pGIz¤, pGEz¤
Ins_EcoRI-ccdB_R	taatgctcttcaTTCCCATCCGTCGCCCGGGCGTGT	Addition of an EcoRI restriction site into ccdB ORF; mutagenesis	pGCz¤	pGIz¤
Ins_EcoRI-ccdB_REagI	taatgctcttcaTTCCCATCCGTCGgCCGGGCGTGT	Addition of an EcoRI restriction site into ccdB ORF; mutagenesis	pGεz¤	pGEz¤
13Mtp_pGGz-1518sqcing<	AACAGGGGCTGGTGAAATG	Sequencing		
14Mtp_pGGz-1296sqcing>	GGCCATATCGGTGGTCATC	Sequencing		
BsaI-sqcingREV	TCCCCAGAACATCAGGTTAATG	Sequencing		
ccdB>_Seq	AGAATGAAGCCCGTCGTCTG	Sequencing		

Table S3. Efficiency, PCR product size and segment position of the real-time PCR primer sets used to quantify Seg-1, -2, -3 and -7 of BTV-1 or BTV-26. Linear regressions were generated from serial plasmid dilutions (10-fold) real-time PCR and efficiency was calculated from the slope of the linear regression equation.

**BTV-# Seg-#**	**BTV-1 Seg-1**	**BTV-26 Seg-1**	**BTV-1 Seg-2**	**BTV-26 Seg-2**	**BTV-1 Seg-3**	**BTV-26 Seg-3**	**BTV-1 Seg-7**	**BTV-26 Seg-7**
Primers	MB286/MB432	MB437/MB438	MB288/MB289	MB273/MB274	MB294/MB295	MB441/MB442	MB296/MB297	MB281/MB282
Effiency (%)	93.9	90.8	91.1	90.0	91.4	98.0	97.9	95.3
PCR product size (bp)	124	152	115	148	126	179	195	125
Segment position	3294-3417	3286-3438	1333-1447	459-606	1254-1379	1770-1948	611-805	643-767
Linear regression slope	-3.4785	-3.5655	-3.5560	-3.5865	-3.5475	-3.3700	-3.3725	-3.4410
R-squared($R^{2)}$	0.9985	0.9962	0.9982	0.9977	0.9993	0.9967	0.9995	0.9965

Table S4. Codon usage for Seg-2 of BTV-1, -4, -6, -9 and -26. Triplets in bold are optimal codons for *Culicoides sonorensis* (based on 21,241 CDS). Colours of the cells are according to a heat map from green (less frequent) to red (most frequent) codon.

Triplet	Amino acid	Frequency					
		BTV-1	BTV-4	BTV-6	BTV-9	BTV-26	Culicoides
**TTT**	F	64	61	49	67	58	63
TTC	F	36	39	51	33	42	37
TTA	L	29	20	27	21	30	26
**TTG**	L(s)	22	27	23	34	24	28
CTT	L	11	9	13	9	8	15
CTC	L	8	11	2	7	7	10
CTA	L	20	19	22	18	14	8
CTG	L(s)	10	13	13	11	16	13
**ATT**	I	35	24	35	22	42	47
ATC	I	27	31	28	37	21	26
ATA	I	38	45	37	40	37	27
ATG	M(s)	100	100	100	100	100	100
**GTT**	V	31	36	40	35	30	31
GTC	V	12	7	13	10	11	20
GTA	V	31	27	21	25	24	22
GTG	V	26	30	26	30	34	27
TCT	S	15	23	13	15	10	13
TCC	S	11	11	5	6	7	13
**TCA**	S	17	18	32	32	24	31
TCG	S	20	18	25	20	17	12
AGT	S	20	18	12	17	27	19
AGC	S	17	12	13	11	15	13
CCT	P	20	34	21	27	9	23
CCC	P	13	9	17	19	12	13
**CCA**	P	42	26	33	32	47	47
CCG	P	25	31	29	22	32	16
ACT	T	18	26	25	17	30	22
ACC	T	24	15	10	20	8	17
**ACA**	T	29	21	35	22	20	44
ACG	T	29	38	31	41	42	16
GCT	A	25	32	30	25	12	28
GCC	A	4	14	7	13	12	18
**GCA**	A	26	22	23	19	37	43
GCG	A	45	32	40	44	39	11
**TAT**	Y	62	59	61	70	69	60
TAC	Y	38	41	39	30	31	40
TAA	*	0	0	100	0	0	36
TAG	*	0	100	0	100	100	15
**TGA**	*	100	0	0	0	0	49
**CAT**	H	69	75	54	68	67	59
CAC	H	31	25	46	32	33	41
**CAA**	Q	47	44	38	43	45	72
CAG	Q	53	56	62	57	55	28
**AAT**	N	59	50	65	68	40	65
AAC	N	41	50	35	32	60	35
**AAA**	K	54	48	43	65	46	69
AAG	K	46	52	57	35	54	31
**GAT**	D	69	66	75	71	65	68
GAC	D	31	34	25	29	35	32
**GAA**	E	51	47	49	49	60	73
GAG	E	49	53	51	51	40	27
**TGT**	C	54	59	67	67	39	58
TGC	C	46	41	33	33	61	42
TGG	W	100	100	100	100	100	100
CGT	R	15	13	18	16	14	14
CGC	R	7	13	12	10	13	6
CGA	R	20	11	5	21	17	20
CGG	R	12	14	9	10	13	8
**AGA**	R	28	25	27	22	29	35
AGG	R	19	24	29	22	14	17
GGT	G	27	31	31	36	30	26
GGC	G	10	17	17	13	20	17
**GGA**	G	35	42	31	33	28	44
GGG	G	29	10	21	18	22	13
